# Nature‐Inspired Innovation in Electrical Engineering Technologies and Applications

**DOI:** 10.1002/advs.202512250

**Published:** 2025-11-17

**Authors:** Ming Li, Anran Mao, Qingwen Guan, Yang Xu, Chang Li, Gang Lu, Eduardo Saiz

**Affiliations:** ^1^ Centre of Advanced Structural Ceramics Department of Materials Imperial College London London SW7 2AZ UK; ^2^ Department of Data and Systems Engineering The University of Hong Kong Hong Kong 999077 China; ^3^ Department of Fibre and Polymer Technology KTH Royal Institute of Technology Teknikringen 56 Stockholm 100 44 Sweden; ^4^ School of Chemistry University of Glasgow Glasgow G12 8QQ UK; ^5^ Department of Electromechanical Engineering Faculty of Science and Technology University of Macau Macau SAR 999078 China; ^6^ Innovation Center for Textile Science and Technology Donghua University Shanghai 201620 P. R. China; ^7^ Department of Chemical and Biomolecular Engineering University of Pennsylvania Philadelphia PA 19104 USA

**Keywords:** adaptive robotics, biomimetic design, electrical engineering, energy harvesting, multimodal sensing

## Abstract

In the rapidly evolving field of science and technology, biomimetic design has emerged as a transformative force in electrical engineering. Leveraging insights from natural evolution, biomimetic methodologies significantly enhance equipment performance and overall system efficiency. This review explores several key functional mechanisms, such as multimodal sensing, energy conversion, and adaptive drive, and showcases state‐of‐the‐art applications. These include biomimetic sensors and detection systems that mimic natural entities like human epidermis, arachnid receptors, and the complex eyes of insects; actuation and robotic systems inspired by the flexible limbs of octopuses, the versatility of elephant trunks, and the cooperative dynamics of ant colonies; as well as renewable energy technologies derived from plant photosynthesis and microbial energy processes, illustrating their potential to transcend traditional engineering boundaries. This biomimetic design not only advances sensor technology, energy harvesting, and adaptive robotics but also holds revolutionary potential for neuromorphic computing and advanced information processing systems. Additionally, the integration of artificial intelligence in these domains, along with their applications in healthcare, environmental monitoring, and human–computer interaction, is discussed. This work underscores the critical integration of natural inspirations with modern engineering to enhance performance and sustainability, offering insights into the future of biomimetic design in electrical engineering.

## Graphical abstract

This review investigates how nature‐inspired design principles revolutionize electrical engineering by translating biological mechanisms, such as sensing, actuation, energy conversion, and neural processing, into advanced intelligent technologies. Through interdisciplinary integration, biomimetic strategies enable efficient, adaptive, and sustainable systems, bridging natural evolution with engineered innovation and redefining the frontier of intelligent, eco‐conscious electrical systems.

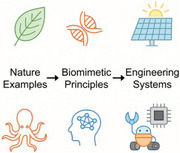

## 1 Introduction

From the advent of vacuum tubes to the development of solid‐state electronics, integrated circuits, and specialized software‐driven systems, electrical engineering has undergone a remarkable evolution.^[^ ^1^ ^]^ These innovations have not only expanded application ranges and enhanced humans’ ability to manage and manipulate electrical signals but have also precipitated transformative changes across nearly every sector of modern industry. As environmental challenges intensify and resource pressures mount, the demand for sustainable, efficient solutions has become increasingly urgent. In response, biomimetic design has emerged as a pioneering field, harnessing nature's time‐tested strategies to address pressing needs in electrical engineering.^[^ ^2^, ^3^, ^4^ ^]^

Nature has refined a variety of complex and efficient functions over billions of years, including self‐healing, multimodal sensing, and ultralow‐power signal processing within neural networks. These evolutionarily optimized functions offer valuable insights into structural and organizational frameworks that could fundamentally transform current electrical engineering systems.^[^ ^5^, ^6^, ^7^, ^8^ ^]^ Biomimetic design represents a paradigm shift, treating organisms as conceptual blueprints for materials, system architectures, and operational strategies (Box 1) —also described as the “bionic revolution” in electrical engineering. This approach merges evolutionary wisdom with cutting‐edge technology, fostering more sustainable practices and groundbreaking innovations.

**Figure 1:**
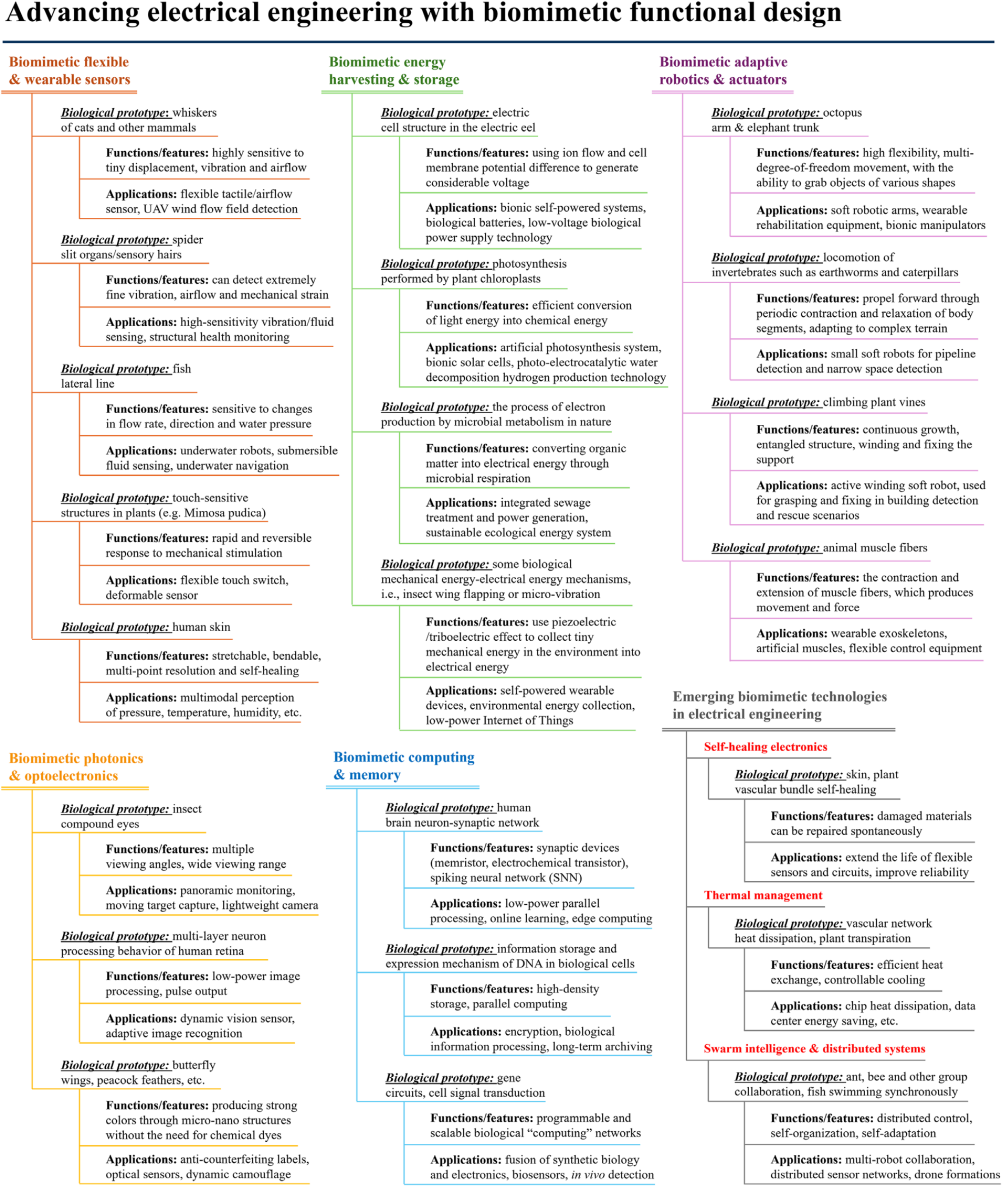
Applications of biomimetic design in electrical engineering. This figure provides a comprehensive overview of the diverse applications of biomimetic design within critical domains of electrical engineering. It illustrates the incorporation of biomimetic principles in flexible wearable sensors (mouse whisker inspired,^[^ ^9^, ^30^, ^31^ ^]^ spider slit receptor inspired,^[^ ^32^, ^33^ ^]^ fish lateral line inspired,^[^ ^34^, ^35^ ^]^ mimosa inspired,^[^ ^48^ ^]^ skin inspired^[^ ^27^, ^28^, ^29^ ^]^), energy harvesting and storage devices (electric eel inspired,^[^ ^173^ ^]^ plant chloroplasts inspired,^[^ ^155^ ^]^ microbial metabolism inspired,^[^ ^177^ ^]^ biological mechanical energy‐electrical energy mechanism inspired^[^ ^150^ ^]^), robotic systems and actuators (octopus inspired,^[^ ^83^ ^]^ elephant trunks inspired,^[^ ^84^ ^]^ earthworm inspired,^[^ ^85^ ^]^ climbing plant vines inspired,^[^ ^86^ ^]^ muscle fibers inspired^[^ ^87^ ^]^), optoelectronic devices (insect compound eyes inspired,^[^ ^22^, ^36^, ^37^ ^]^ human retina inspired,^[^ ^58^ ^]^ structural color inspired^[^ ^63^ ^]^), and advanced computing and storage solutions (neuron‐synaptic network inspired (Intel's Loihi chip,^[^ ^193^ ^]^ IBM's TrueNorth system^[^ ^194^ ^]^), and DNA inspired^[^ ^201^, ^207^ ^]^). Additionally, it highlights other innovative bionic structural and functional designs. Each category is inspired by specific biological prototypes, leveraging their inherent functions and advantages to improve electrical engineering solutions. This strategic integration not only boosts the performance of devices but also fosters novel pathways for technological innovation in the industry.

In an era when sustainable development is a global imperative, biomimetics offers unique and potent solutions for electrical engineering (**Figure** **1**). For example, sensors inspired by the sensory capabilities of animals can detect subtle environmental changes;^[^ ^9^, ^10^, ^11^ ^]^ electrical energy platforms mimicking photosynthesis operate with remarkable efficiency;^[^ ^12^, ^13^, ^14^ ^]^ and biomimetic electrical robots,^[^ ^15^, ^16^, ^17^, ^18^ ^]^ designed after the actuation patterns of plants and animals, support versatile applications across multiple degrees of freedom and scenarios. Further, breakthroughs extend to neuromorphic chips that replicate human brain functions for adaptive, low‐power computing and storage,^[^ ^19^, ^20^, ^21^ ^]^ and to innovative cameras modeled after the compound eyes of insects, enhancing panoramic imaging and environmental monitoring in applications, such as autonomous driving and intelligent surveillance.^[^ ^22^ ^]^ Additionally, advances in synthetic biology and computational modeling enable the simulation and redesign of organisms' molecular mechanisms, producing devices with specialized functions and minimal ecological impact.^[^ ^23^ ^]^

This review comprehensively explores biomimetic design in electrical engineering, focusing on the latest advancements across five key themes: biomimetic sensing and detection systems, biomimetic actuation and robotics, biomimetic energy conversion and storage, biomimetic computing and data storage, and auxiliary biomimetic functional structures that enhance device reliability and environmental adaptability. It emphasizes the transformative potential of biomimetic design to reshape the future of electrical engineering.

## 2 Biomimetic Sensing and Detection Systems

In rapidly developing fields, such as the Internet of Things, innovative healthcare, and environmental monitoring, sensors are core components for information collection. Their performance indicators, sensitivity, response speed, flexibility, and power consumption, are crucial to the system's overall intelligence.^[^ ^24^, ^25^, ^26^ ^]^ Conventional sensor technologies often face challenges in capturing subtle signals, adjusting dynamic ranges, and integrating into flexible wearables. To address these limitations, researchers have engineered various biomimetic sensors inspired by biological sensory systems. These include the tactile sensitivity of human skin,^[^ ^27^, ^28^, ^29^ ^]^ the tactile hairs of mice,^[^ ^9^, ^30^, ^31^ ^]^ microcracks in spider feet,^[^ ^32^, ^33^ ^]^ the lateral line system in fish,^[^ ^34^, ^35^ ^]^ and the compound eyes of insects,^[^ ^22^, ^36^, ^37^ ^]^ etc. (**Table** **1**). These innovative designs have enabled breakthroughs in sensors with high sensitivity, flexibility, self‐repair capabilities, and even multimodal detection, forging new paths for applications across electrical engineering, medical health, and environmental monitoring fields.^[^ ^38^, ^39^, ^40^ ^]^

**Table 1:** Summary of representative biomimetic sensors inspired by natural biological sensory systems.

Biological inspiration	Mechanism/feature mimicked	Materials system	Key performance metrics	Refs.
Human skin (tactile sensitivity)	Pressure‐dependent piezo/piezoresistive/capacitive response	MXene‐textile, iontronic gels, CNT/elastomer	Sensitivity typically 10–600 kPa^−1^, response time ≈20–100 ms, range 0–60 kPa (textile MXene: 652 kPa^−1^, 36/20 ms resp./rec.)	[27, 28, 29]

Mouse whiskers/mammalian vibrissae (tactile hair)	Bending‐induced strain at follicle/base, contact/flow vibrotactile sensing	FBG whiskers, triboelectric whiskers, polymer whiskers	Subtle contact/flow detection, self‐powered tribo versions, used for surface/edge and underwater sensing	^[^ ^9^, ^30^, ^31^ ^]^
Spider slit organs (microcrack sensing)	Crack opening/closing → resistance jump (GF↑)	AgNW/PI, graphene/PU, microcracked thin films	Gauge factor from 10^3^ up to >10⁴ (extreme cases), wide dynamic range, high sensitivity to tiny strains	[32, 33]

Fish lateral line (flow sensing)	Hair‐cell‐like deflection by flow	PVDF‐based hydrogel pillars, IPMC cilia	Velocity detection limits down to 0.23–0.7 mm·s^−1^ (water oscillatory/dipole flow)	^[^ ^34^, ^35^ ^]^
Insect compound eyes (wide‐FOV photodetection)	Microlens array for multidirectional light & wide FOV	Curved microlens + photodiodes (incl. perovskite)	Wide FOV (often >120–180°), angular resolution typically on the order of a few degrees depending on pixel/optics	[22, 36, 37]

### 2.1 Flexible and Wearable Sensors

**Figure 2:**
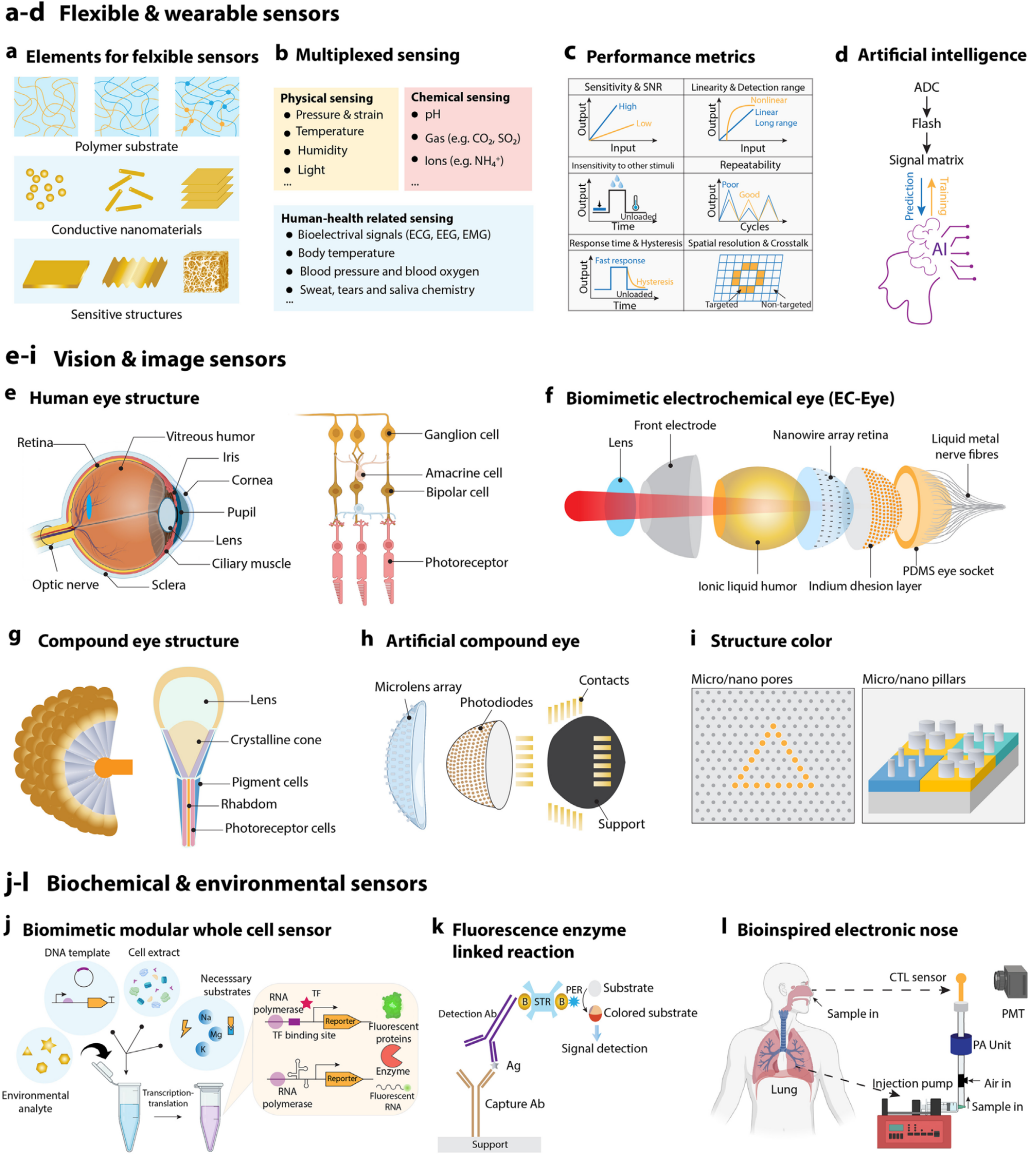
Biomimetic approaches in sensing and detection systems. a) Flexible sensor components: diagram detailing the various elements that make up flexible sensors. (Mouse whisker inspired,^[^ ^9^, ^30^, ^31^ ^]^ spider slit receptor inspired,^[^ ^32^, ^33^ ^]^ fish lateral line inspired,^[^ ^34^, ^35^ ^]^ mimosa inspired,^[^ ^48^ ^]^ skin inspired^[^ ^27^, ^28^, ^29^ ^]^). Part of the figure is created in BioRender. M. A. (2025) https://BioRender.com/j69i992. b) Sensing capabilities: types of sensory inputs that current flexible sensors can detect. (Physical: pressure, strain, temperature, humidity, light, Chemical: pH, gas, ions, Human‐health related: bioelectrical signals (ECG, EEG, EMG), body temperature, blood pressure, blood oxygen, sweat, tears and saliva chemistry).^[^ ^7^ ^]^ c) Performance characteristics: key performance metrics and characteristics typical of flexible sensors. d) AI integration: illustration of how AI modules are embedded in flexible sensors to facilitate data training and predictive analytics.^[^ ^54^ ^]^ e) Single chamber eye structure: depiction of a simple, single chamber eye analogous to basic optical systems. Created in BioRender. M. A. (2025) https://BioRender.com/j69i992. f) Electrochemical eye: inspired by the human eye, this model showcases an electrochemical sensor mimicking ocular functions.^[^ ^58^ ^]^ g) Compound eye structure: structural representation of the multifaceted eyes found in insects and crustaceans. h) Bionic compound eyes: advanced design of synthetic compound eyes for enhanced visual sensing.^[^ ^22^, ^36^, ^37^ ^]^ i) Bionic structural colors:^[^ ^62^, ^63^ ^]^ visual effects created by altering the size and shape of micro‐nano pillars and micropores. j) Cell‐free biosensor system: schematic of the components and response mechanism of a biosensor system to environmental pollutants. k) Fluorescence enzyme‐linked reaction: diagram illustrating the process of a fluorescence enzyme‐linked reaction used for detection.^[^ ^75^, ^76^ ^]^ l) Bionic nose: conceptual design of a sensor system modeled after human olfactory sensory mechanisms.^[^ ^78^ ^]^ Created in BioRender. M. A. (2025) https://BioRender.com/j69i992.

In the realm of wearable electronic devices (smartwatch, fitness tracker, smart clothing, etc.), sensors are essential for being flexible, low‐power, and highly responsive to minute stimuli.^[^ ^24^ ^]^ Over the past decade, advances in nanostructures and flexible film technologies have driven significant progress in the development of biomimetic electronic skin sensors (e‐skin).^[^ ^41^ ^]^ Biomimetic electronic skin (e‐skin) technologies are inspired by the intricate structure and sensory functions of human skin, which includes the epidermis, dermis, and subcutaneous layers. The epidermis contains mechanoreceptors, such as Merkel cells and Meissner's corpuscles, that detect light touch and pressure.^[^ ^42^ ^]^ The dermis, which is abundant in sensory nerve endings like Pacinian and Ruffini corpuscles, plays a key role in sensing deeper pressures, vibrations, and skin stretching.^[^ ^43^ ^]^ These biological mechanisms are replicated in e‐skin by embedding conductive nanomaterials into flexible polymer substrates, enhancing their sensitivity and responsiveness to external stimuli. This biological model has been central to the evolution of e‐skin, leading to the creation of ultrathin, flexible sensing membranes by incorporating graphene, carbon nanotubes, and metal nanowires into polymer matrices. (**Figure** **2**a).^[^ ^44^ ^]^ These membranes translate external changes in pressure, temperature, humidity, and airflow into measurable electrical signals, such as resistance, capacitance, and voltage (Figure 2b,c). Flexible pressure and strain sensors based on 2D materials have broad applications in medical health monitoring and wearable electronics. Studies have demonstrated that MXene nanosheet and porous polyester fiber composites can be used to fabricate breathable pressure sensors, offering high sensitivity (652.1 kPa^−1^), a broad detection range (0–60 kPa), and rapid response/recovery times (36/20 ms), making them ideal for real‐time blood pressure measurement and pulse detection.^[^ ^45^ ^]^ In the case of strain sensing, an exemplary system is a self‐powered flexible sensor modeled after ion gradient generators. The LiCl‐FP material generates a high‐humidity region, while the exposed yarn creates a low‐humidity region, resulting in an ion gradient that generates a voltage or current. This sensor can measure strain from 0.5% to 100%, maintaining good repeatability over 1000 cycles, and has been demonstrated in applications, such as respiration monitoring.^[^ ^46^ ^]^

In addition to skin‐like multifunctional sensors, researchers have developed a range of bioinspired sensors that replicate the specific sensing characteristics of organisms, thereby extending the functionality of artificial sensing systems across diverse environments. For instance, drawing inspiration from the tactile sensing abilities of mouse whiskers, a lightweight, self‐powered biomimetic mouse whisker sensor (BMWS) combines triboelectric and structural design to enable sophisticated environmental detection. The system excels at stable collision detection, direction identification, hole‐width recognition, real‐time distance measurement, and intelligent early warning. When integrated with an artificial neural network (ANN), the BMWS effectively differentiates between ground and spatial objects, showcasing its potential for autonomous exploration and environmental sensing.^[^ ^9^ ^]^ Similarly, the dynamic shape adjustment of bat‐wing membranes in response to airflow has inspired a capacitive feedback system based on the microspring effect of dielectric elastomers (DEs).^[^ ^47^ ^]^ This system, which integrates hair‐like pressure sensors at the leading edge, uses a feedback–feedforward control mechanism to enable real‐time lift regulation and disturbance compensation. This bioinspired aerodynamic sensing technique improves airflow detection sensitivity and flight stability, holding great promise for smart aerodynamic structures, autonomous aerial systems, and wind‐field monitoring. In addition, nature's mechanical sensing mechanisms, such as those found in spider slit receptors and the mimosa plant, have inspired the creation of flexible sensors capable of detecting minute changes in resistance or current caused by vibrations, deformation, or pressure through surface microcracks or responsive microstructures.^[^ ^48^ ^]^ A notable example is the meta‐crack flexible sensor, which utilizes an innovative crack‐opening mechanism to detect minute deformations with exceptional accuracy. It achieves an extraordinarily high gauge factor (>1000 at a strain of 10^−4^) on substrates with a Poisson's ratio of −0.9 and offers a strain resolution of 10^−5^.^[^ ^33^ ^]^ Additionally, inspired by the lateral line system (LLS) in fish and amphibians, which allows them to detect subtle variations in water flow, researchers have created a hydrodynamic artificial velocity sensor (HAVS) made from a P(VDF‐TrFE)/BTO electrospun nanofiber mat integrated onto a PI substrate with circular cavities and a PMMA cilium.^[^ ^35^ ^]^ This composite nanofiber mat demonstrates improved crystallinity and piezoelectric properties, achieving a velocity detection limit of 0.23 mm s^−1^, outperforming traditional flow sensors. The HAVS's directional sensing ability further highlights its potential for real‐time hydrodynamic monitoring and underwater robotics. Together, these bioinspired technologies illustrate how natural sensing mechanisms, from whiskers and wings to cracks and lateral lines, can be effectively adapted into advanced artificial sensors with unparalleled sensitivity, versatility, and multifunctionality, paving the way for intelligent systems capable of detecting and responding to complex environments.

Biomimetic wearable sensors have emerged as a game‐changer in health monitoring, enabling simultaneous tracking of multiple physiological signals (Figure 2b). These include electrocardiograms, electroencephalograms, electromyography, blood pressure, body temperature, and respiratory rates.^[^ ^39^, ^49^ ^]^ For example, a bioinspired multilayered E‐skin (SPRABE‐skin) provides accurate monitoring of ECG, EMG, and EEG signals with exceptional precision. It is characterized by its softness (3.36 MPa), breathability, and outstanding sensitivity (gauge factor = 63 494 at 485% strain), making it suitable for wireless, long‐term physiological signal detection in comprehensive healthcare monitoring.^[^ ^50^ ^]^ Moreover, some biomimetic wearable sensors could also continuously monitor skin moisture, electrolyte imbalances, and gas volatiles, facilitating chronic disease management and providing early warnings for acute medical events.^[^ ^7^, ^39^ ^]^ The capture and transmission of bioelectric signals are facilitated by conductive components within the flexible sensors, such as salt ions, conductive nanomaterials, and conductive polymers. Meanwhile, biophysical signals, such as respiratory rate, body temperature, blood pressure, and pulse are converted into electrical signals, along with resistance, capacitance, and potential, with enhanced sensitivity compared to conventional sensors. For example, a polyvinyl alcohol‐polyacrylamide (PVA‐PAM) double‐network hydrogel sensor doped with LiCl and MXene exhibits high humidity sensitivity (−103.4%/%RH) over 40%–85% RH, along with remarkable stretchability (>3000%) and excellent environmental stability. Due to the strong hydrogen bonding and chemical cross‐linking, it facilitates real‐time monitoring of respiration and sleep, providing a durable solution for flexible humidity sensing in healthcare applications.^[^ ^51^ ^]^ Additionally, monitoring of various body fluid components relies on the changes in electrochemical signals triggered by reactions between specific sensor components and the target analytes. Further integrating them with wireless communication technologies, such as Bluetooth, NFC, or ultrawideband, allows for real‐time data upload to the cloud. Users can then receive personalized health management insights through artificial intelligence and big data analysis (Figure 2d).^[^ ^52^ ^]^ This multimodal approach, coupled with advanced wireless and cloud‐based technologies, has proven highly effective in practical applications, such as athlete training, rehabilitation medicine, and remote health monitoring.^[^ ^53^, ^54^ ^]^ For example, a motion recognition sensor combining electromagnetic‐triboelectric composite electrodes with a magnetic PVA–Fe_3_O_4_ fiber membrane allows for real‐time tracking and identification of motion patterns. The system achieves 99.58% accuracy in recognizing five different motion types through machine‐learning‐based signal analysis, providing an efficient solution for athlete training, rehabilitation, and remote health monitoring.^[^ ^54^ ^]^ In addition, with the integration and advancement of self‐powered technologies, such as triboelectric nanogenerators and thermoelectric converters, we have seen breakthroughs in truly “stick‐on, use‐now” wearable monitoring systems in multiple application scenarios.^[^ ^38^, ^55^ ^]^

### 2.2 Vision and Image Sensors

In biological visual systems, single‐chamber eyes and compound eyes each display unique structural and functional characteristics tailored to the specific needs of different organisms. Single‐chamber eyes, predominantly found in vertebrates (including humans), boast a straightforward yet practical design with a single optical cavity. Central features of this type include a convex lens that precisely focuses light onto a photosensitive retina at the back of the eye (Figure 2e), providing high‐resolution, sharp vision crucial for accurately perceiving the external environment.^[^ ^56^, ^57^ ^]^ In addition, when the eyes see a blurry object, the brain and visual system receive signal feedback that the retinal image is blurred, and the ciliary muscle adjusts the curvature of the lens until the image on the retina becomes clear; the automatic focus system used in the camera imitates the ability of animal eyes to adapt to focal length. Drawing inspiration from the human eye, researchers have developed an electrochemical eye (EC‐Eye) that replicates this biological structure. The EC‐Eye features a glass lens that directs light onto a hemispherical nanowire retina, designed to replicate human photoreceptors, facilitating image reconstruction and high‐resolution visual sensing (Figure 2f). The nanowire‐array retina closely mimics the structure of the human eye and shows promise for visual prosthetics, with potential for improved resolution and signal processing.^[^ ^57^, ^58^ ^]^ In contrast, compound eyes, typical of arthropods like insects and crustaceans, consist of hundreds to thousands of small, independent photosensitive units known as ommatidia. Each ommatidium functions as a separate photoreceptor, contributing to the compound eye's ability to capture a panoramic view (Figure 2g).^[^ ^59^ ^]^ Unlike traditional cameras, which suffer from imaging distortion and field of view limitations due to their planar lenses and linear sensor arrays, the spherical arrangement of ommatidia in insect compound eyes allows for nearly 360° ultrawide‐angle imaging and rapid dynamic response. Emulating this natural design, researchers employ micro‐nano fabrication techniques to create microlens arrays on spherical caps or hemispherical substrates (Figure 2h), significantly enhancing both the imaging quality and the field of view.^[^ ^22^, ^36^, ^37^ ^]^ This biomimetic compound eye camera technology markedly reduces edge distortion, and is particularly effective in applications requiring expansive real‐time monitoring, such as in drones, autonomous vehicles, and security systems.^[^ ^60^ ^]^ It can also accurately detect high‐speed objects, with capabilities reaching up to 9120 frames per second.^[^ ^22^ ^]^

The structural color effects observed in organisms, such as butterfly wings, beetle shells, and peacock feathers not only create visually striking appearances but also inspire the development of tunable optical devices/sensors based on micro‐ and nanostructures (Figure 2i).^[^ ^61^ ^]^ By altering the size, shape, and refractive index of micro‐nano columns or microholes, these devices can selectively respond to specific wavelengths of light based on diffraction/Bragg effect, resonance effects, interference/thin‐film effect, etc. These changes in structural color are reversible and respond dynamically to variations in external pressure, temperature, or chemical environments, enabling significant transformations in the optical signal.^[^ ^62^, ^63^, ^64^ ^]^ For example, bioinspired structural color electronic skins (SC E‐skins) made from liquid metal particles, colloidal crystal arrays, and ultraelastic hydrogels display remarkable integrated performance, including over 1100% elongation, a gauge factor of 3.26, a response time of ≈100 ms, durability for more than 1500 cycles, and accuracy (*R* ^2^ > 99.5%). These combined electrical‐optical sensors offer high stretchability, sensitivity, and stability, making them highly promising for next‐generation smart wearable health‐monitoring devices.^[^ ^63^ ^]^ Such features are particularly advantageous for security and anticounterfeiting applications, where the unique “fingerprints” of micro–nano structures provide a reliable means of authentication.^[^ ^61^, ^65^ ^]^ Furthermore, when integrated with neuromorphic chips (to be introduced in Section 5.1), these devices can preprocess and extract features of optical information at the hardware level, rather than transferring all the data to the central processor. This integration significantly reduces the computational load and power consumption of subsequent processing stages. In addition to structural coloration, bioinspired color‐changing systems that replicate the chromogenic mechanisms of cephalopods and chameleons have emerged as a powerful method for dynamic optical modulation in response to external stimuli.^[^ ^66^ ^]^ These systems alter their skin color through reversible changes in pigment cell shape and reflective nanostructures. A notable example is an artificial chameleon device that combines a thermochromic liquid‐crystal layer with vertically aligned silver nanowire heaters.^[^ ^67^ ^]^ This multilayer configuration allows for precise, high‐resolution color modulation by controlling local temperatures, overcoming the pixelation challenges of traditional camouflage systems. With integrated sensors and feedback mechanisms, the device can swiftly adjust its surface color to match its environment, enabling smooth, reversible optical transitions and providing a complete device‐level artificial camouflage system with natural adaptive capabilities. Another example, inspired by the multispectral camouflage abilities of cephalopods,^[^ ^68^ ^]^ features a flexible bi‐functional skin that combines active cooling and heating layers with a thermochromic surface. This design enables continuous color modulation across both visible and infrared spectra using simple temperature adjustments. The scalable, pixelated design allows for localized, autonomous color changes, enabling adaptive camouflage that blends with intricate background patterns. This system demonstrates wearable, device‐level visible‐to‐infrared concealment, merging biological inspiration with soft, multispectral electronic skins.

### 2.3 Biochemical and Environmental Sensors

In the field of biochemical and environmental sensing, cell‐free biosensing technology has attracted significant attention for its remarkable sensitivity and specificity.^[^ ^69^, ^70^ ^]^ Throughout their long evolutionary history, microorganisms have developed various molecular response mechanisms to detect specific environmental substances. Leveraging this natural capability, researchers have extracted the molecular machinery (transcription, translation system, or enzyme system, etc.) in the microorganisms’ cells, retaining the enzymes and factors required for transcription and translation, and then built a reaction system together with synthetic/designed DNA, RNA, protein, and other sensor elements.^[^ ^71^ ^]^ Furthermore, advances in synthetic biology now enable the design and construction of highly sensitive and specific modular cell‐free sensors through genetic engineering.^[^ ^72^ ^]^ These sensors are adept at detecting hazardous substances like heavy metals, antibiotics, and pesticides (Figure 2j). When the target molecule is present, it triggers transcription, translation, or enzymatic reactions in the in vitro system, resulting in detectable signals, such as fluorescence, luminescence, or color change.^[^ ^73^ ^]^ Another notable advancement is the incorporation of bioluminescence mechanisms from fireflies or deep‐sea luminescent organisms into microfluidic chips. This technology enables rapid detection of trace molecules or toxins by measuring the light emitted during the chemical reaction of substrates with fluorescent proteins and luciferases (Figure 2k).^[^ ^74^ ^]^ Leveraging the high throughput capabilities of microfluidics, bioluminescence has demonstrated its unique potential in fields, such as drug screening, molecular diagnostics, and personalized medicine.^[^ ^75^, ^76^ ^]^ Additionally, electronic noses, inspired by the human olfactory system, utilize a diverse array of gas sensors combined with machine learning algorithms to achieve highly sensitive detection of volatile organic compounds or toxic gases (Figure 2l).^[^ ^77^, ^78^ ^]^ In particular, a portable e‐nose equipped with a TMKFF–1D‐CNN–LSTM fusion model demonstrated highly accurate prediction of gas mixtures (SO_2_, NO_2_, CO). The model achieved R^2^ values of 0.93, 0.98, and 0.99, with RMSE values of 0.0760, 0.0711, and 3.3825, and MAE values of 0.0507, 0.0549, and 2.5874, respectively, showcasing outstanding performance, particularly in detecting low concentrations of SO_2_.^[^ ^78^ ^]^ If these biomimetic electronic noses are adapted into flexible and wearable formats, they could also provide real‐time monitoring and early warning of harmful gases in workplaces or disaster sites.

In summary, biomimetic sensing and detection technologies are evolving from mere “inspiration and reference” to “deep interdisciplinary integration.” This progression integrates novel material fabrication, micro‐ and nanoprocessing, and advanced algorithms, offering versatile solutions for intelligent healthcare, environmental conservation, and industrial automation. In wearable devices, trends toward flexibility, self‐healing, high sensitivity, and multimodal sensing are becoming the norm, revolutionizing personal health management and acute disease monitoring. In the realm of vision and image sensing, natural phenomena, such as insect compound eyes and structural colors have expanded the imaging capabilities and spectral responses of cameras. Additionally, in biochemical and environmental detection, advancements in cell‐free sensing and bioluminescence technologies have propelled the development of “molecular‐level” sensitive detection systems. Looking ahead, future research should focus on enhancing energy efficiency and accuracy in sensing technologies through biomimetic design, particularly by advancing self‐powered sensors and integrating edge computing. Moreover, natural organisms use a limited range of materials—both organic and inorganic—to construct highly complex sensors. Understanding how organisms overcome material constraints through structural optimization informs the core concept behind our development of the next generation of biomimetic sensors. By integrating this biological approach with a diverse selection of artificial materials, we aim to enhance functionality beyond the capabilities of natural biological sensors. This strategic combination allows us to transcend natural limitations and innovate sensors that can detect and measure phenomena previously beyond biological sensing capabilities. To elevate the intelligence of sensor systems, interdisciplinary collaborations combining artificial intelligence and big data analysis should be encouraged. Such efforts are poised to drive transformative changes across smart cities, precision medicine, and clean energy sectors.

## 3 Biomimetic Actuation and Soft Robotics

When it comes to robotics, traditional rigid mechanical systems excel in precision but often struggle with flexibility in unstructured, complex environments, and do not perform well with delicate, brittle items.^[^ ^79^ ^]^ Biomimetic robotics technology is progressively overcoming these challenges by emulating the adaptable and flexible movements of natural organisms.^[^ ^3^, ^80^, ^81^, ^82^ ^]^ Inspired by biological’ actuation mechanisms such as the tentacles of octopuses,^[^ ^83^ ^]^ elephant trunks,^[^ ^84^ ^]^ earthworms,^[^ ^85^ ^]^ climbing plant vines,^[^ ^86^ ^]^ and muscle dynamics^[^ ^87^ ^]^ (**Table** **2**); as well as the collective behavior of ant colonies and bird flocks, researchers have made significant advances. They have developed a diverse array of soft robots, artificial muscles, and distributed control systems for robot swarms.^[^ ^25^, ^88^, ^89^, ^90^ ^]^ These innovations introduce an unprecedented level of flexibility and intelligence to the field of robotics, enhancing their capability to navigate and operate effectively in dynamic settings.

**Table 2:** Summary of representative biomimetic actuators and soft robotic systems.

Biological inspiration	Actuation mechanism	Materials/system	Actuation performance	Refs.
Octopus arm	Pneumatic actuation + on‐body liquid metal network that mimics the functionality of an octopus nerve	Liquid metal circuit pattern +silicone	It can endure up to 710% uniaxial and 270% biaxial strain, enabling it to autonomously operate within confined spaces.	[83]

Elephant trunks	Temperature actuation	Liquid crystal elastomer (LCE)‐based multifiber	The liquid crystal elastomer (LCE) employed in this study exhibits an estimated shear modulus of ≈473 kPa, and when integrated with embedded copper coils, it demonstrates reversible contraction strains of around −33% under thermal stimulation.	^[^ ^84^ ^]^
Earthworm	Pneumatic actuation	Silicone rubber of different Shore hardness, with a soft Shore 00–50 (Ecoflex 00–50, Smooth‐On)	For the high‐density triangular grid, expanding the actuation stroke from 15% to 30% boosts the movement speed from 0.5 to 0.9 mm s^−1^. However, pushing the stroke further to 45% yields minimal improvement (around 1 mm s^−1^) and may reduce actuator durability.	[85]

Climbing plant vines	Pneumatic eversion+ electromagnet steering unit	Elastomer+ electromagnet unit	Generate a magnetic force exceeding 10 N, allowing precise steering across eight spatial directions.	^[^ ^86^ ^]^
Human skeletal muscle	Electromagnetic actuation	Magnetized hard magnet+ elastomeric structure+ electrical coil intertwined with soft magnetic spheres	Delivers a high output force (≈210 N kg^−1^), a substantial contraction ratio (up to 60%), fast actuation at 60 Hz, and low‐voltage functionality (<4 V), all within a durable and compact design.	[87]

### 3.1 Soft Robots and Artificial Muscles

**Figure 3:**
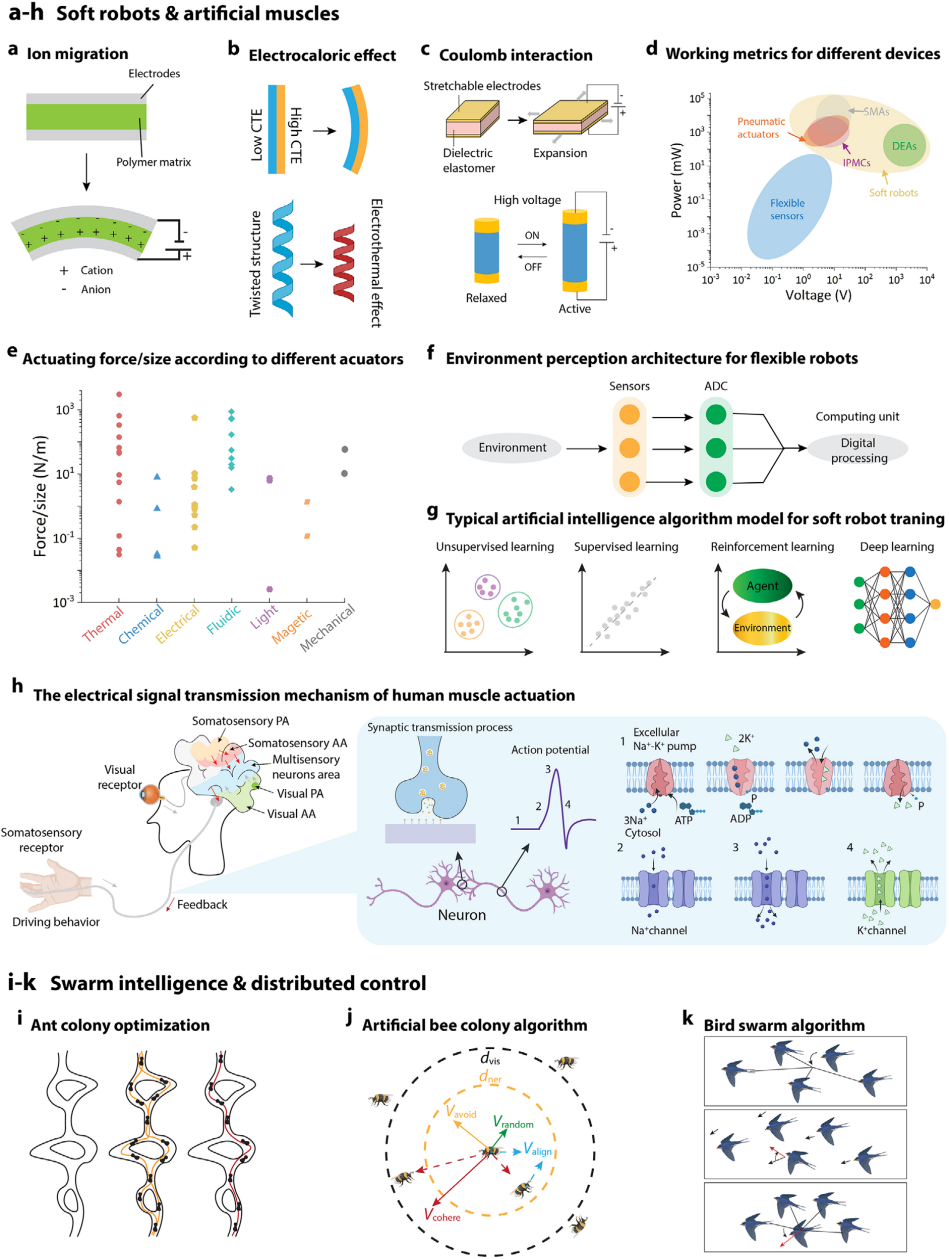
Biomimetic approaches in soft actuation and robotics. a) Conductive hydrogel: schematic diagram of actuation achieved by ion migration within a conductive hydrogel.^[^ ^98^ ^]^ b) SMP/SMA deformation: schematic representation of shape memory polymers/alloys (SMP^[^ ^103^ ^]^/SMA^[^ ^101^ ^]^) undergoing deformation induced by the electrothermal effect. c) Electroactive polymers:^[^ ^105^ ^]^ schematic diagram of electroactive polymers driven by Coulomb force generated from charge accumulation on electrodes after voltage application. d) Power consumption comparison: comparative analysis of driving power consumption across various soft robotic devices. Adapted with permission.^[^ ^26^ ^]^ Copyright 2023, American Chemical Society. e) Force output versus size comparison: graphical comparison of output force relative to size among soft robots with different actuation types. Adapted with permission.^[^ ^80^ ^]^ Copyright 2024, Springer Nature. f) Environmental perception: schematic diagram showcasing how the soft robot achieves the perceiving and interacting with its environment. g) AI algorithm model:^[^ ^25^, ^80^, ^125^ ^]^ typical model of artificial intelligence algorithms used for training soft robots, enhancing their decision‐making and learning capabilities. h) Human muscle actuation: schematic representation of human muscle actuation, highlighting the continuous adjustment of actions through feedback from sensory organs to achieve precise control. Part of the figure is created in BioRender. M. A. (2025) https://BioRender.com/j69i992. i) Ant colony foraging algorithm:^[^ ^131^ ^]^ schematic diagram of the bionic ant colony foraging algorithm, illustrating how robotic systems can mimic ant foraging behavior to optimize routes. j) Artificial bee colony algorithm:^[^ ^132^ ^]^ schematic diagram outlining the artificial bee colony algorithm, used by robots for efficient decision‐making and resource location. Created in BioRender. M. A. (2025) https://BioRender.com/j69i992. k) Bird swarm algorithm:^[^ ^133^ ^]^ schematic diagram of the bird swarm algorithm, demonstrating how robotic systems can emulate the complex, adaptive behaviors of bird flocks to enhance cooperative tasks. Created in BioRender. M. A. (2025) https://BioRender.com/j69i992.

Electrically actuated soft robots employ flexible materials, such as hydrogels, shape memory polymers/alloys (SMP/SMA), and electroactive polymers (EAPs), enabling multidegree‐of‐freedom movement without rigid skeletons. This mimics the dexterity of octopus tentacles navigating tight spaces or elephant trunks grasping objects.^[^ ^89^, ^91^, ^92^, ^93^, ^94^, ^95^, ^96^ ^]^ In hydrogel‐based actuators, ion migration induced by an external electric field alters the local electrostatic field and osmotic pressure, leading to asymmetric swelling and bending (**Figure** **3**a).^[^ ^97^ ^]^ For instance, an electroosmosis‐driven hydrogel actuator, created through layer‐by‐layer assembly of cracked metal nanoparticle electrodes via amphiphilic interactions, achieved a strain of over 20% and an energy density of 1.06 × 10⁵ J m^−3^, about ten times greater than that of skeletal muscle or conventional electrochemical actuators.^[^ ^98^ ^]^ This actuator demonstrated various motions, such as two‐degree‐of‐freedom bending and spring‐out actions, highlighting its potential for high‐precision soft robotics.

Shape memory alloys and polymers operate through electrothermal phase transitions that induce bending, twisting, or contraction (Figure 3b).^[^ ^99^ ^]^ In SMAs, reversible martensite–austenite transformations enable structural recovery,^[^ ^100^ ^]^ as exemplified by a fabric‐like actuator that combines SMA springs with electrostatic clutches (ESClutches). This hybrid design generates 40 N of force from only 18 g of material at over 35% strain. It sustains deformation with just a few milliwatts of power, cutting energy consumption by more than 70% compared with standalone SMAs.^[^ ^101^ ^]^ Conversely, shape memory polymer (SMP)‐based actuators use dual‐component systems in which one phase stores elastic deformation, while the other adjusts stiffness in response to heat or stress.^[^ ^102^ ^]^ A multimorph dielectric elastomer actuator (DEA) integrated with SMP fibers and stretchable heaters achieved reversible, programmable morphing behavior, Joule heating reduced fiber rigidity by two orders of magnitude, allowing precise modulation between soft and stiff regions. This actuator exhibited a tip deflection of over 300° at 5 kV and a blocking force of 27 mN, enabling multiaxis bending and complex reconfiguration, such as adaptive gripping.^[^ ^103^ ^]^

Electroactive polymers, including ionic polymer–metal composites (IPMCs) and dielectric elastomers (DEAs), provide another class of electrically responsive actuators capable of fast, muscle‐like contractions (Figure 3c).^[^ ^104^ ^]^ A compact columnar dielectric elastomer actuator (DEA) made from VHB acrylic film and carbon black electrodes demonstrated multidegree actuation, with 50° bending and 13% axial strain. Integrated sensing capabilities allowed for real‐time feedback, enabling precise tasks, such as grasping and screwing.^[^ ^105^ ^]^ While IPMCs function at only a few volts, their response is constrained by ion migration; incorporating nanoscale channels and aligned polymer networks can notably enhance ion transport and minimize actuation delay.^[^ ^106^ ^]^ In contrast, DEAs respond in milliseconds and provide high strain and energy density but typically require voltages above 1 kV (Figure 3d).^[^ ^107^ ^]^

In addition to electrical actuation, soft robots also utilize fluidic (pneumatic or hydraulic),^[^ ^108^, ^109^, ^110^, ^111^ ^]^ magnetic,^[^ ^112^, ^113^ ^]^ thermal (via direct heat sources, electrothermal, and photothermal effects),^[^ ^114^, ^115^, ^116^ ^]^ chemical (influenced by factors like humidity, pH, and ion concentration),^[^ ^117^ ^]^ and mechanical^[^ ^15^, ^118^ ^]^ stimuli to achieve movement and adaptability across various environments. These can range from pneumatic grippers handling delicate objects to magnetically controlled capsules used in pipeline inspection and biomedical applications.^[^ ^3^ ^]^ Nevertheless, electrically actuated systems still fall short of pneumatic and hydraulic counterparts in terms of force output and power density (Figure 3e).^[^ ^80^ ^]^ To address this limitation, researchers are exploring hybrid electro‐hydraulic actuators and high‐dielectric elastomers to boost energy density while maintaining flexibility.^[^ ^119^, ^120^ ^]^ The integration of multiple actuation strategies and advanced material technologies will ultimately enable soft robots to strike an optimal balance of power, precision, and adaptability across biomedical, industrial, and exploratory fields.

As soft robots increasingly tackle more complex tasks and enter diverse application areas, there is a growing demand for their autonomy and “intelligence.” A pivotal goal in soft robotics research is to develop an “intelligent” robot with self‐perception capabilities, enabling it to sense its environment, interact effectively, and make autonomous decisions (Figure 3f).^[^ ^121^ ^]^ This requires the robot to continuously gather and process information about itself and its surroundings, dynamically adjusting its movements to achieve situational awareness and autonomous control. Recent advances in integrating artificial intelligence (AI) algorithms with soft robot control systems have been significant, especially with the incorporation of machine learning and deep learning techniques to address challenges in soft robot modeling and control (Figure 3g). Given the inherent complexities of soft structures, such as high nonlinearity in mechanical and electrical signals, large deformations, and hysteresis, traditional physical modeling and control strategies often fall short of accurately capturing their behavior.^[^ ^122^ ^]^ To overcome these limitations, researchers are employing supervised, unsupervised, and reinforcement learning methods, enabling robots to “learn” their dynamic characteristics directly from data rather than relying solely on precise mathematical models.^[^ ^123^, ^124^ ^]^ For instance, by processing sensor inputs through advanced neural networks (such as convolutional neural networks (CNNs) and recurrent neural networks (RNNs)), soft robots can convert complex sensory information into specific actuator responses, facilitating effective closed‐loop control.^[^ ^25^, ^80^, ^125^ ^]^ This learning‐based approach allows robots to continually adjust and correct their movements, enhancing precision and robustness. In high‐stakes environments, such as minimally invasive surgery or operations in confined spaces, AI‐enabled controllers on soft robots can fine‐tune movements based on real‐time feedback from sensors, such as deformation gauges, force sensors, or visual cues, minimizing excessive force and deviations from intended paths.^[^ ^126^, ^127^, ^128^ ^]^ This control architecture mirrors the biological mechanisms in humans, in which muscle movements are initiated by bioelectric signals from the brain, with continuous adjustments based on feedback from sensory organs, ensuring precise, controlled actions (Figure 3h).^[^ ^39^ ^]^ Soft robots are progressively embodying this sensor‐decision‐execution loop, empowered by AI, to adapt to their environments and make autonomous decisions.^[^ ^129^, ^130^ ^]^ For example, a research team developed a flexible collaborative robot arm driven by pneumatic artificial muscles with a bio‐inspired antagonistic tendon structure at the joints.^[^ ^126^ ^]^ Equipped with tension sensors, this robot arm can be manually taught through direct manipulation, relying on its internal sensors to achieve high‐precision repetitive motions with path repetition accuracy errors less than 0.3% of the body length and a hysteresis of less than 2%. This demonstrates that with sophisticated sensing and control design, soft structures can meet the precision and stability requirements of industrial applications, while inherently ensuring safety.

### 3.2 Swarm Intelligence and Distributed Control

Swarm intelligence draws inspiration from the collaborative behaviors observed in natural organisms, such as ants, bees, and bird flocks. It leverages simple local communications and rules among numerous individual robots to cultivate a collective intelligence that significantly surpasses the capabilities of any single robot. For instance, ants secrete pheromones to mark their trails, leading other ants to follow these highly concentrated pheromone paths and efficiently discover the shortest routes (Figure 3i).^[^ ^131^ ^]^ This biological principle has been effectively adapted for path planning in robotic swarms. Similarly, bees utilize a dance to communicate the location of nectar sources to their hive mates (Figure 3j).^[^ ^132^ ^]^ This method of communication has been translated into strategies for collaborative decision‐making within groups of robots. Moreover, each bird in a flock adjusts its movements according to simple local rules to interact with its neighbors, enabling the entire group to navigate and adapt in complex, dynamic environments (Figure 3k).^[^ ^133^ ^]^ This principle has been employed to advance the development of intelligent and autonomous groups of robots. These mechanisms, borrowed from nature, not only improve the environmental adaptability of robots but also offer fresh insights into the smart control and decision‐making processes within complex systems.

As distributed control and reinforcement learning algorithms have matured, researchers have enabled autonomous coordination and flexible cooperation among hundreds of small drones. These capabilities include obstacle avoidance, path planning, task allocation, and formation reconstruction.^[^ ^134^, ^135^, ^136^, ^137^, ^138^, ^139^ ^]^ Typically, each drone is equipped with basic computing capabilities and multimodal sensors, allowing it to make independent decisions based on data from neighboring drones and environmental cues, and to share this information within the swarm. Such capabilities ensure that drone swarms are highly efficient and robust, particularly for complex, uncertain tasks, such as disaster response and marine environmental monitoring. For instance, MIT's Computer Science and Artificial Intelligence Laboratory developed the “RoboBees” robots, which mimic the behavior and collaborative prowess of bees to perform complex tasks, such as environmental surveillance and search‐and‐rescue operations.^[^ ^140^ ^]^ Notably, if an individual drone fails, the swarm can automatically reconfigure its formation or substitute the malfunctioning unit based on real‐time feedback, enhancing the group's overall stability and adaptability. Furthermore, integrating machine learning models with multimodal data sources, including visual, infrared, and ultrasound sensors, enables these robotic groups to identify targets, discern terrain structures, and dynamically plan movement paths. The distributed AI algorithms employed in these systems, such as domain adaptive learning, ensemble learning, and multiagent systems, are crucial for optimizing group collaboration and improving decision‐making speed and accuracy.^[^ ^25^ ^]^ Domain adaptive learning allows robots to adjust to new environmental dynamics swiftly, ensemble learning enhances decision accuracy by integrating data from multiple sensors, and multiagent systems permit individual robots to make independent decisions in response to environmental changes while maintaining effective communication across the swarm.

While bioinspired actuation and soft robotics technologies hold tremendous potential, they also face significant challenges, including limited material lifespan and durability, high energy consumption, and complex control systems. Soft materials often suffer from fatigue due to prolonged, repeated deformations. Artificial muscles need breakthroughs, including further biomimetic hierarchical structures and directional arrangements to improve mechanical properties, and biomimetic ion channels to enhance ion transport rates, to improve their response speed and power density. Bandwidth limitations and real‐time performance requirements constrain the distributed communication and coordination required for swarm intelligence. Future research should concentrate on developing and optimizing new materials, advancing control algorithms, and enhancing the efficiency of collaboration among multirobot systems. These efforts aim to forge a more adaptable, energy‐efficient, and intelligent robotic platform. The future development direction of biomimetics in this field mainly includes: 1) System‐level bionics, from single functions (biomimetic sensing, biomimetic actuation) to overall system level (actuation, perception, communication, decision‐making). Comprehensive biomimetics enable soft robots to exhibit greater robustness, flexibility, and intelligence in real environments. 2) Comprehensive biological inspiration, integrating multiple bionic concepts—such as hierarchical structure (extending service life), muscle‐like actuation (improving efficiency), group intelligence (distributed collaboration), can bring about a leap in overall performance, not just the optimization of local functions. Such advancements will support critical applications in medical rehabilitation, environmental monitoring, industrial automation, and beyond, pushing the boundaries of what these innovative technologies can achieve.

## 4 Biomimetic Energy Conversion and Storage

As global energy demand escalates and environmental protection challenges become more acute, the development of efficient, clean energy solutions has become imperative.^[^ ^141^, ^142^ ^]^ Nature offers exemplary models of high efficiency in energy conversion and storage, such as photosynthesis,^[^ ^143^ ^]^ bioelectricity generation in electric eels,^[^ ^144^ ^]^ and the metabolic reactions of microorganisms,^[^ ^145^ ^]^ etc. Biomimetic energy conversion technology draws inspiration from natural processes to efficiently convert solar, mechanical, biomass, and chemical energy. These innovations not only advance self‐powered systems but also promote sustainable practices in electrical engineering by converting wasted energy into usable electricity through various biomimetic energy conversion devices, offering fresh perspectives on green energy solutions.^[^ ^146^, ^147^ ^]^

### 4.1 Triboelectric Nanogenerators

**Figure 4:**
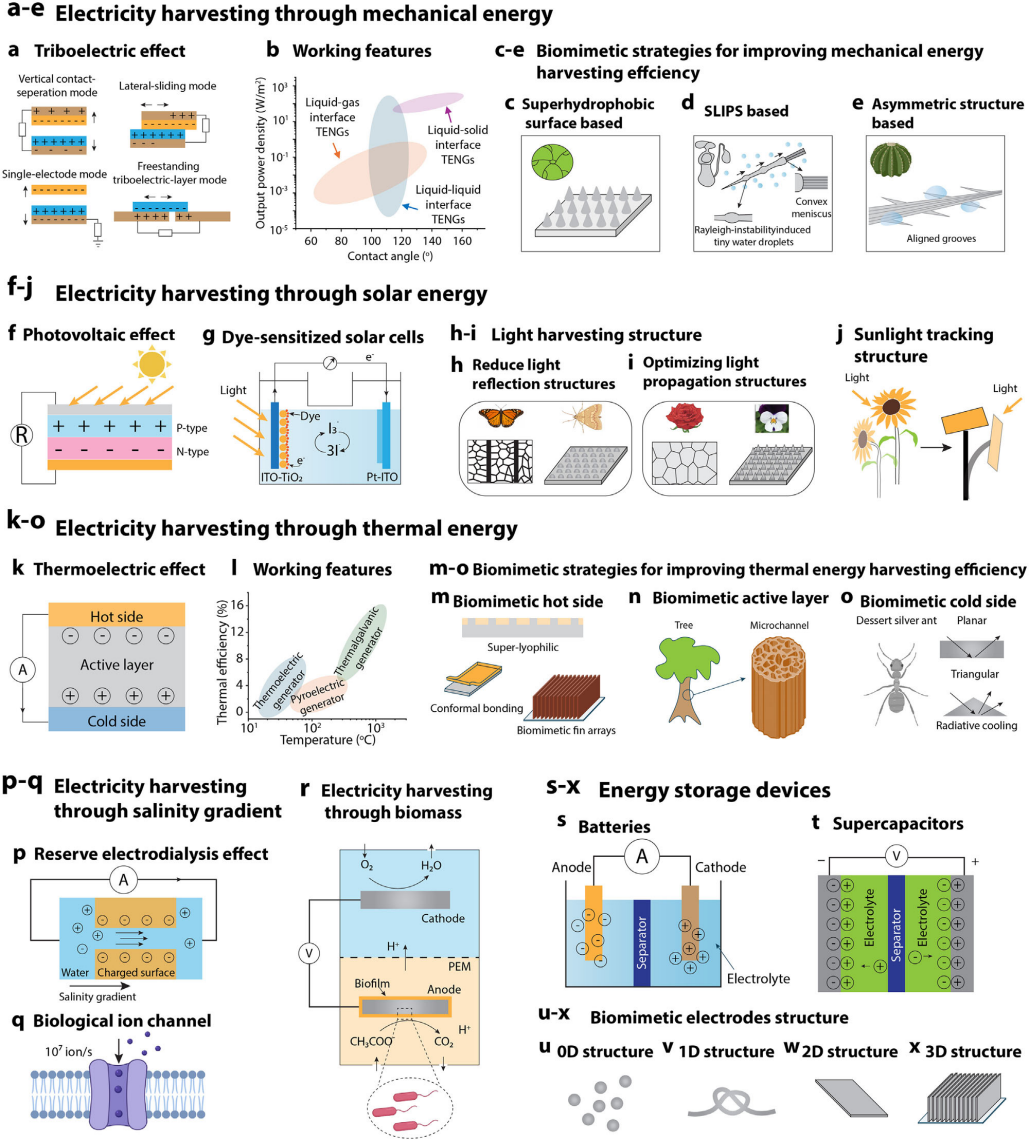
Biomimetic approaches in energy conversion and storage. a) Schematic representation of the four operational modes of the triboelectric nanogenerator (TENG). b) Graph depicting the contact angles and output power densities of TENGs across various working interfaces. Adapted with permission.^[^ ^146^ ^]^ Copyright 2024, Springer Nature. c–e) Biomimetic strategies for improving mechanical energy harvesting efficiency of TENG. c) Illustration of a TENG surface with a lotus leaf‐like superhydrophobic structure designed for kinetic energy collection from water droplets.^[^ ^150^ ^]^ d) TENG surface featuring a Nepenthes‐inspired liquid‐infused structure, aimed at preventing liquid‐liquid contact on dielectric surfaces.^[^ ^152^ ^]^ e) Diagram of a cactus‐like TENG surface structured to actively harvest atmospheric moisture, utilizing interface charge separation for energy generation.^[^ ^153^ ^]^ f) Photovoltaic effect in solar cells. g) Functional mechanism of a dye‐sensitized solar cell.^[^ ^155^ ^]^ h–i) Biomimetic light harvesting structures (moth eye^[^ ^159^ ^]^ and black butterfly wing^[^ ^158^ ^]^) on solar cells. h) Biomimetic design on solar cell surfaces to minimize light reflection. i) Biomimetic design on solar cell surfaces (rose petals^[^ ^160^ ^]^ and viola flowers^[^ ^157^ ^]^) to optimize light propagation across solar cell surfaces. j) Schematic of an adaptive solar cell capable of dynamically tracking natural light.^[^ ^161^ ^]^ The viola flower picture in Panel (i). Reproduced with permission.^[^ ^157^ ^]^ Copyright 2017, American Chemical Society. k) Schematic of the thermoelectric effect. l) Performance metrics of thermoelectric generators, detailing working temperatures and thermal efficiencies across different mechanisms (thermoelectric, pyroelectric, and thermogalvanic generators). Adapted with permission.^[^ ^146^ ^]^ Copyright 2024, Springer Nature. m–o) Biomimetic strategies for improving thermal energy harvesting efficiency of thermoelectric generators. m) Biomimetic design of the hot end structure in thermoelectric generators (enhances liquid–solid interfacial wetting,^[^ ^163^ ^]^ conformal attachment,^[^ ^164^ ^]^ and hierarchically structured fins^[^ ^165^ ^]^). n) Biomimetic design of the active layer in thermoelectric generators.^[^ ^166^ ^]^ o) Biomimetic design of the cold end structure in thermoelectric generators (Saharan silver ants inspired^[^ ^169^ ^]^ and Archaeoprepona Demophon inspired^[^ ^170^ ^]^). p) Diagram illustrating the reverse electrodialysis effect for harvesting energy from salinity gradients.^[^ ^174^ ^]^ q) Schematic of a biological ion channel. Created in BioRender. M. A. (2025) https://BioRender.com/j69i992. r) Working mechanism of microbial fuel cells.^[^ ^177^ ^]^ s–x) Energy storage devices. s) Batteries t) Supercapacitors. u–x) Biomimetic electrode structures employed in batteries and supercapacitors, u) 0D structure,^[^ ^185^ ^]^ v) 1D structure,^[^ ^186^ ^]^ w) 2D structure,^[^ ^187^ ^]^ x) 3D structure.^[^ ^188^ ^]^

The triboelectric nanogenerator (TENG) is an innovative technology that leverages the triboelectric effect observed in nature, such as the interaction between skin and plant leaves. Utilizing contact electrification and electrostatic induction between different materials (**Figure** **4**a), TENG efficiently converts mechanical energy from the environment, such as vibration, pressure, friction, and droplet kinetic energy, into electrical energy.^[^ ^148^, ^149^ ^]^ Through biomimetic design (mimicking natural surfaces) that optimizes the microstructure of material surfaces, TENG significantly enhances charge separation efficiency and increases the contact area, thereby boosting triboelectric charge density per unit area (Figure 4b). For instance, mimicking the micro‐ and nanoscale protrusions on superhydrophobic lotus leaves (Figure 4c) alters the interaction of water droplets with the surface, thereby enhancing charge separation. This bionic approach enables the TENG to generate, on average, three times the electrical energy as traditional hydrophobic surface TENGs when processing droplets at an impact frequency of 165 Hz.^[^ ^150^ ^]^ Additionally, inspired by the slippery characteristics of Nepenthes, TENGs utilizing slippery liquid‐infused porous surfaces (SLIPS) technology effectively eliminate liquid–liquid contact on dielectric surfaces (Figure 4d). This avoids unnecessary wetting transitions and increases the liquid–solid contact area, improving the efficiency of droplet kinetic energy collection.^[^ ^151^ ^]^ The nanogenerators, based on lubricating layer designs, can sustain a stable output voltage of up to 40 V under continuous droplet impact.^[^ ^152^ ^]^ Beyond directly harvesting kinetic energy from droplets, specific bioinspired structures can also capture atmospheric moisture to drive energy collection through interface charge separation mechanisms. For example, a self‐driven TENG (Figure 4e), emulating cactus thorns, demonstrated exceptional power output by capturing clean water from the atmosphere and converting the mechanical energy of falling droplets into electrical energy, achieving an open‐circuit voltage of up to 103.2 V.^[^ ^153^ ^]^

### 4.2 Solar Energy Conversion

Plants efficiently convert solar energy into chemical energy through chloroplasts, playing a crucial role in ecosystem balance. Artificial photosynthesis technology seeks to emulate this natural process by transforming solar energy into storable forms such as electricity. Recent advancements in materials science and manufacturing technology have significantly advanced solar cells (Figure 4f), transitioning from traditional crystalline silicon to perovskite solar cells (PSCs) and evolving from single‐ to multistructured or tandem configurations, thereby consistently surpassing existing efficiency benchmarks.^[^ ^154^ ^]^ The biomimetic design of solar cells encompasses multilevel innovations across function, interface, and structure. Functionally, plants' light‐collecting antennas in natural photosynthesis capture sunlight and generate electrons, which are then transferred along an electron transport chain to facilitate energy conversion.^[^ ^143^ ^]^ Dye‐sensitized solar cells (DSSCs, Figure 4g) replicate this mechanism using molecular dyes that absorb light and produce electrons, which are injected into TiO_2_ and transferred to Pt‐indium tin oxide (ITO) electrodes to reduce the redox agent in the electrolyte, achieving a photoconversion efficiency (PCE) of up to 13%.^[^ ^155^ ^]^ Interface biomimicry focuses on optimizing the solar cell surface design to enhance sunlight collection. This involves the incorporation of layered micro/nanotextured structures inspired by moth eyes and butterfly wings, which reduce light reflection and enhance light capture (Figure 4h), alongside multiscale structures inspired by rose petals and violet flowers that optimize light propagation and interaction, boosting solar energy absorption (Figure 4i).^[^ ^156^, ^157^ ^]^ For instance, the micro‐ and nanostructures on the wings of the black butterfly significantly enhance sunlight harvesting from a wide range of angles. Drawing inspiration from these structures, nanostructured thin photovoltaic absorbers with irregular nanoholes were developed, resulting in a 90% improvement in absorption at normal incidence and a 200% increase at larger angles, highlighting their potential for thin‐film solar cells.^[^ ^158^ ^]^ Additionally, moth‐eye structures were fabricated on polycarbonate (PC) substrates using R2P UV‐NIL, achieving an average reflection of 1.21% in the visible spectrum (380–760 nm) at normal incidence and less than 4% reflection at 50°.^[^ ^159^ ^]^ Furthermore, sunlight‐harvesting designs inspired by the microstructures of rose petals^[^ ^160^ ^]^ and viola flowers^[^ ^157^ ^]^ resulted in improvements in power conversion efficiency (PCE) of 13.7% and 6.2%, respectively, compared to flat, nontextured solar cells. Moreover, inspired by sunflowers, researchers have developed adaptive solar cells that dynamically sense and respond to variations in natural light. These cells maintain stable energy conversion efficiency while significantly improving energy collection, particularly under oblique illumination angles. This technology, based on phototropic behavior, can improve solar energy harvesting by up to 400% compared to conventional nontropic materials (Figure 4g).^[^ ^161^ ^]^ These biomimetic approaches not only improve the functionality of solar cells but also contribute to the sustainability of energy systems.

### 4.3 Thermal Energy Conversion

In human society, a substantial amount of energy is lost as heat. Effectively recovering this energy could significantly mitigate the greenhouse effect and the energy crisis.^[^ ^142^ ^]^ Current thermoelectric conversion technologies, including thermoelectric generators, pyroelectric generators, and thermogalvanic generators (Figure 4k). The operating temperature ranges and thermal efficiency of these devices are detailed in Figure 4l.^[^ ^162^ ^]^ The bionic design of thermoelectric conversion systems encompasses three main aspects: heat transfer design at the hot end, efficient ion transport in the active layer, and heat dissipation at the cold end.^[^ ^146^ ^]^

For the hot‐end design, the goal is to maximize both the thermal contact area and the overall heat‐transfer efficiency between the heat source and the thermoelectric generator (Figure 4m). Different biomimetic strategies are adopted depending on the physical state of the heat source. A heat‐conduction‐enhanced hydrovoltaic power generator is demonstrated for liquid heat sources, combining a flexible ionic thermoelectric gelatin with a porous dual‐size Al_2_O_3_ hydrovoltaic generator.^[^ ^163^ ^]^ The system improves liquid–solid interfacial wetting, boosting the water evaporation rate and enhancing output voltage. This leads to a stable open‐circuit voltage of 6.4 V, the highest reported thus far, by effectively converting solar energy into thermal energy while maintaining a consistent temperature difference across the thermoelectric generator. In cases where the heat source is solid, bioinspired flexible adhesive interfaces, reminiscent of gecko toe pads or octopus suckers—enable conformal attachment, effectively minimizing interfacial thermal resistance and eliminating insulating air gaps. For instance, a wearable thermoelectric generator (TEG) has been developed with bioinspired, flexible adhesive interfaces that mimic the structure of gecko toe pads, ensuring secure, conformal attachment. This design reduces interfacial thermal resistance and removes insulating air gaps, thereby improving heat transfer. The system achieves an impressive open‐circuit voltage of 1 V cm^−^^2^ at a temperature difference of 95 K. It also incorporates a wavelength‐selective metamaterial film on the cold side, significantly enhancing performance under solar irradiation, making it highly suitable for wearable energy harvesting during outdoor activities.^[^ ^164^ ^]^ For gaseous heat sources, hierarchically structured fins or hedgehog‐like needle arrays are introduced to increase the effective heat‐exchange surface area and promote fluid turbulence, leading to improved heat dissipation. In particular, a porous pin‐fin design optimized using the NSGA‐II‐TOPSIS method resulted in a 22.89% increase in system output power (P) and an 82.98% reduction in pressure drop (Pd). The optimal configuration, featuring densely arranged downstream porous pin fins, enhanced the ETEG system's performance, achieving a maximum efficiency index of 5.86 W Pa^−1^ with two porous pin fins.^[^ ^165^ ^]^ In the active layer design, inspiration is drawn from the hierarchical vascular and fiber network of a tree, which facilitates directional mass and heat transport. The resulting design introduces ordered pore or channel arrays that provide continuous pathways for ion and heat transfer. Such structural alignment increases the specific surface area, shortens the diffusion distance, and improves the coupling between thermal and ionic conduction, collectively enhancing the overall conversion efficiency (Figure 4n). For instance, a cellulosic membrane with oxidized cellulose molecular chains achieves a thermal gradient ratio of 24 mV K^−1^, more than twice the previous record.^[^ ^166^ ^]^ This enhancement results from the insertion of sodium ions into type II cellulose, yielding a flexible, biocompatible heat‐to‐electricity conversion device with significant potential for large‐scale production. Regarding the cold‐end design, cues are taken from the Saharan silver ant, an organism capable of maintaining sub‐body temperatures even under extreme desert heat.^[^ ^167^ ^]^ Bioinspired radiative‐cooling materials derived from this model incorporate triangular or corrugated photonic structures that strongly reflect near‐infrared radiation and efficiently dissipate absorbed heat in arid environments (Figure 4o). Recent advancements in radiative‐cooling materials have expanded the scope of biomimetic thermal energy management, shifting from active heat conversion to passive thermal regulation and energy conservation.^[^ ^168^ ^]^ Inspired by the microstructured cuticle of Saharan silver ants, which combines high solar reflectivity with strong mid‐infrared emissivity, researchers have developed hierarchical photonic coatings and polymer membranes that simultaneously achieve high solar reflectance (>95%) and significant mid‐infrared emissivity in the 8–13 µm atmospheric window.^[^ ^169^ ^]^ These materials enable heat to dissipate to outer space without external power, facilitating subambient cooling of ≈4–6 °C, even in direct sunlight. Moreover, recent studies have extended biomimetic radiative cooling to multifunctional photonic architectures. For example, nanostructures inspired by the reconstructed butterfly wing scales of *Archaeoprepona demophon* combine nanoporous matrices with periodic nanogratings, enabling both radiative cooling (with temperature reductions up to 8.45 °C) and vibrant structural coloration covering more than 90% of the sRGB color gamut. This demonstrates how natural photonic structures can be designed to integrate thermal management with optical functions, opening new possibilities for energy‐saving and aesthetically functional material systems.^[^ ^170^ ^]^ When integrated with active energy‐conversion systems such as photovoltaic cells, thermoelectric modules, and triboelectric nanogenerators, these radiative‐cooling materials create a synergistic solution for reducing heat buildup, stabilizing device output, and enhancing overall energy efficiency. These hybrid architectures showcase the application of natural thermoregulation mechanisms in engineered materials, linking passive cooling with active energy conversion and paving the way for sustainable, low‐carbon, and energy‐autonomous technologies. These biomimetic thermal‐management approaches not only improve the performance and longevity of thermoelectric devices but also chart new directions for the development of next‐generation green energy systems.

### 4.4 Osmotic Energy Conversion

At the interface between seawater and river water, the salinity gradient generates substantial osmotic energy, also known as blue energy, with power densities reaching up to 0.8 kWh m^−^^3^ in certain regions.^[^ ^147^ ^]^ Reverse electrodialysis (RED) technology harnesses this gradient by utilizing semipermeable membranes that facilitate ion diffusion between these water bodies to generate electricity. This process is characterized by charge separation and the formation of potential differences (Figure 4p). However, traditional ion‐exchange membranes face challenges in effectively harnessing this energy due to their substantial thickness, limited pore sizes, and susceptibility to fouling.^[^ ^171^ ^]^ Drawing inspiration from the efficient energy‐transfer mechanisms of biological ion channels (Figure 4q), employing high surface charge and optimal pore structures in nanofluidic channels can greatly enhance ion selectivity and flow permeation.^[^ ^172^, ^173^ ^]^ This approach significantly increases the energy conversion power density. For instance, single‐layer MoS_2_ nanofluidic membranes, designed based on the principles of biological ion channels, have been shown to increase the power density of osmotic energy to 10^6^ W m^−^^2^, vastly outperforming traditional ion exchange membranes.^[^ ^174^ ^]^ Moreover, the design of these biomimetic nanofluidic channel membranes features exceptional flexibility and a hierarchical structure, making them highly suitable for large‐scale integrated applications. This provides an efficient and sustainable method for capturing and utilizing osmotic energy, paving the way for advancements in renewable energy technology.^[^ ^175^ ^]^

### 4.5 Microbial Fuel Cells

Microbial fuel cells (MFCs) directly convert the chemical energy of organic matter into electrical energy through microbial metabolism (Figure 4r), offering the benefits of being clean, efficient, and renewable. However, practical applications of MFCs often encounter challenges, such as low electron transfer efficiency, limited microbial load, and obstructed mass transfer processes.^[^ ^145^ ^]^

In response to these issues, researchers are looking to nature for solutions, employing biomimetic design to enhance MFC performance across multiple dimensions. One key area of focus is the electrode materials. For instance, the use of a PPy/NFs/PET anode, which mimics biological hierarchical structures, significantly enhanced the anode's surface roughness. This design not only supports a robust microbial community but also enhances electron‐exchange efficiency, a crucial factor for the effectiveness of bioelectrochemical systems, thereby significantly boosting microbial fuel cell performance. When utilized with *Escherichia coli* as a microbial catalyst, this anode configuration has reached an impressive maximum power density of 2420 mW m^−^^2^.^[^ ^176^ ^]^ Another key focus is the development of the microbial‐electrode interface. For instance, by integrating materials such as nitrogen‐doped carbon nanotubes (N‐CNTs), reduced graphene oxide (rGO), and polyaniline onto the electrode surface, a hierarchically porous composite is formed to replicate the extracellular matrix. This approach promotes biofilm growth and enhances electron transfer, resulting in a peak power density of 1137 mW m^2^ in *S. putrefaciens* CN32 MFCs, which is 8.9 times greater than that of a traditional carbon cloth anode and outperforms other nitrogen‐doped composites.^[^ ^177^ ^]^ Alternatively, a 3D macroporous scaffold design enhances microbial attachment and electron transfer by offering greater internal pore space. This configuration fosters efficient bacterial colonization, resulting in a power density of 1184 mW m^2^, surpassing that of traditional 3D anode materials.^[^ ^178^ ^]^ The open 3D macroporous structure increases the interfacial area, thereby enhancing mass transfer, substrate diffusion, and charge transfer, while maintaining low overpotential (−27 mV) and low internal resistance (7.104 Ω cm^2^), improving both system efficiency and stability.

Additionally, to address bottlenecks in electron and mass transfer, some researchers have drawn inspiration from the hierarchical channel structures of plant xylem (Figure 4n). They developed MMFCs featuring a multilevel flow channel network (converging, straight, diverging), which improves the efficiency of substrate and product exchange. The MMFC featuring the diverging channel (MMFC‐D) achieved a maximum power density of 2447.7 mW m^2^, which is 429% higher than the converging channel and 24% higher than the straight channel, demonstrating the significant influence of microchannel geometry on performance.^[^ ^179^ ^]^ For larger‐scale applications, the design of modular or stacked MFCs incorporates the ecosystem's cascade concept. These systems connect multiple units in series and parallel configurations and are complemented with loops for circulating nutrients and by‐products. Such designs not only adapt flexibly to different pollutant loads but also enhance synergistic efficiency under various operating conditions.^[^ ^180^ ^]^

In summary, by integrating principles of biomimicry, researchers are making substantial progress in addressing the key challenges faced by microbial fuel cells. This approach not only improves electron transmission, microbial load, and mass transfer but also paves the way for broader application of MFCs in fields, such as clean energy production and wastewater treatment.

### 4.6 Energy Storage Devices

In the field of energy, alongside the focus on improving the performance of energy‐harvesting devices, the role of energy storage devices is equally pivotal.^[^ ^181^, ^182^, ^183^ ^]^ Nature provides a vast reservoir of inspiration for the development of advanced surfaces and interfaces with sophisticated functions and complex structures. These innovations are exceptionally vital for renewable energy storage and conversion devices, such as batteries and supercapacitors, where bio‐inspired interfaces are widely used (Figure 4s,t). This section primarily focuses on the structural design of electrode materials, exploring how principles from nature can optimize these critical components. The biomimetic design of electrode interfaces can be categorized into four distinct types based on their dimensional characteristics: 0D, 1D, 2D, and 3D interfaces.^[^ ^181^, ^184^ ^]^

0D biomimetic interfaces typically consist of single atoms, quantum dots, and nanoparticles (Figure 4u). These materials are favored in energy storage and conversion devices due to their highly reactive active sites or functional groups. An example of this is the ferrite electrode, which has been synthesized through biomimetic mineralization. This electrode features an ordered array of 0D interfaces and delivers a power density of up to 4304 W kg^−1^, demonstrating its potential for efficient energy storage and conversion.^[^ ^185^ ^]^

1D bionic interfaces include various structures, such as nanorods, nanotubes, nanowires, and nanofibers. These forms are particularly noted for their rapid charge transfer capabilities and excellent mechanical toughness along the axial direction (Figure 4v). These materials are strategically designed with an axial orientation that optimizes mechanical properties and charge‐transfer efficiency along a specific direction. This design approach mirrors structural principles found in nature, such as the highly oriented configurations of plant cell walls and animal muscle fibers, which naturally exhibit exceptional toughness and mechanical resilience under load. A prime example of these principles in action is seen in polythiophene matrix nanowires used in the supercapacitor. These are intricately coiled into dense, single fibers with a diameter of ≈100 µm, demonstrating the practical application of this biomimetic strategy. This structural arrangement not only ensures sustained electrochemical stability but also provides excellent fatigue resistance. Impressively, these nanowires achieve a capacitance of up to 32.9 mF cm^−2^,^[^ ^186^ ^]^ underscoring their efficiency and durability in demanding applications.

2D bionic interfaces are represented by nanoplates, nanobelts, nanosheets, and nanofilms, which can be produced through self‐assembly methods or artificial manufacturing processes (Figure 4w). Inspired by natural weaving patterns found in vines, bird nests, and spider webs, composite fabric electrodes are created by layering multi‐walled carbon nanotubes (MWCNT) and RGO, which are vacuum‐filtrated onto Ni‐coated cotton fabrics. This structure is used to develop waterproof, wearable power devices that exhibit exceptional mechanical properties, achieving areal capacitances of 6.2 and 3.2 F cm^−2^ after 10 000 charge‐–discharge cycles, with no capacitive decay observed after 10 000 bending tests.^[^ ^187^ ^]^ However, textiles are not the only application for 2D biomimetic structures. Nature's layered assembly and composite strategies also prove invaluable in fields that demand large surface areas, flexibility, and high conductivity.^[^ ^184^ ^]^

3D biomimetic interfaces integrate the structural advantages of the previous dimensions, displaying exemplary interface stability and ventilation capabilities (Figure 4x). A notable construction in this category involves stacking 0D hydrogen‐bonding active sites, 1D carbon nanotubes or nanofibers, and 2D MXene derivatives. For example, a GA@UiO‐66‐NH_2_ hybrid was developed by covalently bonding amine‐functionalized UiO‐66‐NH_2_ to carboxylate‐functionalized graphene (GA) via amide linkages, yielding a hierarchical structure that enhances electrochemical performance. This hybrid achieved a capacitance of 651 F g^−1^, significantly surpassing traditional graphene‐based materials. In an asymmetric supercapacitor using Ti_3_C_2_TX MXene as the opposing electrode, the device achieved a power density of 16 kW kg^−1^ and an energy density of 73 Wh kg^−1^, with 88% capacitance retention after 10 000 cycles.^[^ ^188^ ^]^

This chapter comprehensively reviews the current advancements in biomimetic energy harvesting and storage technologies, highlighting their unique ability to emulate natural energy flows and achieve efficient conversion. Looking to the future, to propel the interdisciplinary integration and overall innovation within biomimetic energy conversion and storage technologies, targeted efforts are crucial in several key areas: 1) Enhance high power and rapid response capabilities: Simulate the efficient ion and electron transport networks found in animals and plants, such as microchannels in muscle fibers or vascular systems in plants. By developing multiporous channels, directionally conductive networks, and biomimetic flow designs, we can significantly improve the power density and rapid‐discharge capabilities of devices. This enhancement is essential for meeting the demands of high‐load applications such as smart grids and transportation systems. 2) Develop multiscale biomimetic interfaces and intelligent responsive materials: Integrate biomimetic concepts from the molecular to the macroscopic level, drawing inspiration from the waterproof micro–nano structures of lotus leaves and the robust fibers of spider silk. This approach will enhance interface compatibility and functional synergy across electrodes, electrolytes, and packaging systems. Additionally, developing materials that can intelligently respond to environmental stimuli such as temperature, pressure, and light will enable dynamic adjustments in the energy conversion process. 3) Achieve deep integration with artificial intelligence: Leverage embedded sensing and data analysis technologies to enable adaptive monitoring and control of energy conversion and storage processes. Utilizing machine learning algorithms to predict and manage charging and discharging processes can help identify and promptly rectify potential faults, thereby maximizing system efficiency and extending lifespan. 4) Develop manufacturing processes for extreme conditions and scalable applications: Explore the resilience and versatility of organisms that thrive in extreme environments, such as polar microorganisms and deep‐sea life. These insights will inform the design of biomimetic energy materials capable of withstanding high temperatures, pressures, or intense radiation. Developing scalable, cost‐effective biomimetic manufacturing processes is essential for translating laboratory innovations into broadly deployable industrial technologies. By intensifying research on material structure design, functional mechanisms, and intelligent control, and by fostering close collaborations with fields, such as artificial intelligence, advanced manufacturing, and emerging industries, biomimetic energy conversion, and storage technologies are poised for transformative advances. These enhancements in efficiency, durability, and scalability will enable the deployment of more viable and sustainable solutions across diverse applications, including portable electronics, wearable devices, and distributed energy systems.

## 5 Biomimetic Computing and Data Storage

With the advent of the Big Data Era and the rapid advancement of artificial intelligence technologies, traditional computing architectures are increasingly challenged by the demands for higher energy efficiency and enhanced parallel processing capabilities. Biomimetic computing and information storage technologies, inspired by the computational mechanisms of biological neural networks and DNA‐based storage methods, are at the forefront of exploring new, efficient, and low‐power data processing and storage solutions.^[^ ^189^, ^190^ ^]^ These technologies offer groundbreaking ideas and solutions that could revolutionize future computing systems, addressing current limitations and setting new benchmarks for performance and sustainability.

### 5.1 Biomimetic Neuromorphic Computing

**Figure 5:**
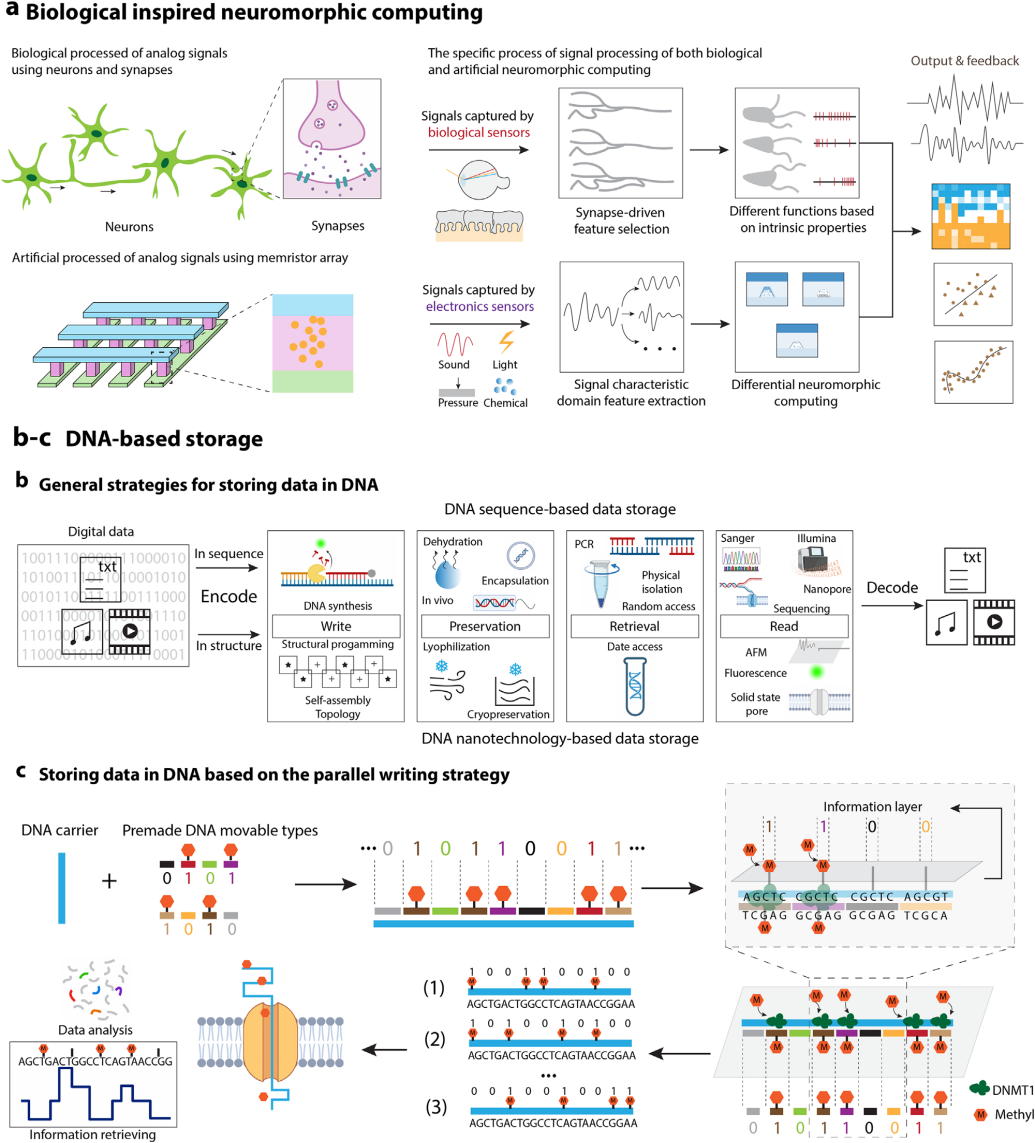
Biomimetic approaches in computing and information processing. a) Comparative schematic illustrating the differential processing method utilized in memristor arrays, alongside biological sensory processing and current neuromorphic processing techniques. b,c) Overview of DNA‐based storage systems, detailing the six critical steps involved: encoding, writing, preservation, retrieval, reading, and decoding. b) Description of two principal strategies for data storage using DNA, including the DNA sequence‐based scheme and the DNA nanotechnology‐based scheme.^[^ ^201^ ^]^ c) Conceptual diagram of a newly proposed DNA‐based data storage system employing a parallel writing strategy, emphasizing efficiency and scalability.^[^ ^207^ ^]^

Biomimetic neuromorphic computing technology, which mimics the structure and dynamic behavior of neurons and synapses at the hardware level, provides an efficient solution for large‐scale parallel processing and adaptive learning (**Figure** **5**a).^[^ ^189^, ^191^, ^192^ ^]^ Compared to traditional von Neumann architectures, this technology significantly reduces energy loss and time delays associated with data transmission. It is highly effective at adapting to complex environmental changes and offers rapid, flexible learning and response capabilities. For instance, Intel's Loihi chip (60 mm^2^, 14 nm process) uses a spiking neural network model with hierarchical connectivity and programmable synaptic learning rules.^[^ ^193^ ^]^ It can solve LASSO optimization problems with a three‐order‐of‐magnitude improvement in energy‐delay product over traditional CPU‐based solvers, demonstrating the advantages of spike‐based computation. Similarly, IBM's TrueNorth system incorporates 4096 neurosynaptic cores, 1 million neurons, and 256 million synapses, consuming only 63 milliwatts while processing 400 × 240‐pixel video input at 30 fps.^[^ ^194^ ^]^ This system is highly scalable and efficient for real‐time applications such as multiobject detection and classification. Furthermore, integrating advanced research in memristors or phase‐change memories enables these systems to simulate multilevel synaptic plasticity with increased precision. Specifically, when combined with hafnium oxide memristors in metal‐oxide‐semiconductor transistors, they facilitate analog vector‐matrix multiplication with arrays of up to 128 × 64 cells, achieving 5–8‐bit precision. With a 99.8% device yield and stable multilevel states, these systems significantly improve the accuracy of continuous input data processing, making them highly efficient for complex computational tasks.^[^ ^195^ ^]^

In practical applications, biomimetic neuromorphic computing technology has achieved significant progress in developing low‐power intelligent edge devices. For example, smart sensors now employ computing‐in‐sensor technology to preprocess image or voice signals locally.^[^ ^196^ ^]^ By performing tasks such as classification or autoencoding directly on the sensor, these systems transmit only essential feature data, drastically reducing the need for data transmission and processing. This approach replicates the brain's sensory processing efficiency and is crucial for real‐time edge computing in domains such as autonomous vehicles and robotics. It is instrumental in wearable medical devices, such as smart ECG monitors. For instance, an ultralow power, reconfigurable biomedical AI processor (BioAIP) has been developed using 65 nm CMOS technology.^[^ ^197^ ^]^ This processor features a flexible architecture capable of handling various AI tasks, an event‐driven design with approximate data compression to conserve power, and an adaptive‐learning architecture to enhance classification accuracy. Applied to tasks like ECG arrhythmia detection, EEG seizure identification, and EMG hand gesture recognition, the processor consumes minimal energy per classification (several microjoules) while maintaining high accuracy, outperforming existing designs optimized for single biomedical AI functions. This BioAIP provides versatility by interfacing with various biomedical analog frontends, supporting a broad range of system applications. Looking ahead, as more complex neural dynamics and reconfigurable 3D chip technologies are combined, bionic neuromorphic computing is poised to unlock even greater potential in fields like general artificial intelligence and high‐performance computing. By more deeply mimicking the brain's sparse connections and pulse signal characteristics at the physical level, the next generation of neuromorphic systems may achieve capabilities such as multimodal real‐time processing, heterogeneous distributed scheduling, and the integration of approximate and precise computing. These advancements could revolutionize edge computing, autonomous driving, and brain–computer interfaces, heralding a new era of innovation in these cutting‐edge domains.^[^ ^198^ ^]^

### 5.2 Artificial DNA‐Based Storage

Artificial DNA storage mimics the mechanisms for genetic information storage in organisms, using artificially synthesized DNA molecules as information carriers. With its ultrahigh storage density and excellent long‐term stability, DNA storage significantly surpasses traditional storage media.^[^ ^199^, ^200^, ^201^ ^]^ The core technology involves converting digital data into nucleotide sequences (A, T, C, G) and encoding them in a “quaternary” form into DNA strands, facilitated by advanced chemical synthesis and sequencing technologies (Figure 5b).^[^ ^202^ ^]^ Stored under low‐temperature and light‐proof conditions, DNA can be preserved stably for centuries, showcasing immense potential for applications requiring long‐term storage, such as geological databases, museum archives, and cloud storage.^[^ ^203^ ^]^

Recent advancements have led to significant breakthroughs in the efficiency of DNA data writing, reading, and security. For example, integrating machine learning algorithms into the analysis of DNA sequences has enabled highly accurate, low‐cost pattern recognition from large DNA datasets.^[^ ^204^ ^]^ This method efficiently uncovers complex relationships between DNA features and their corresponding outcomes, requiring less training data and yielding more precise results compared to traditional techniques. In the realm of data security, a novel multilayer encryption scheme using deoxyribonucleic acid‐reconstructed chaotic sequences (DNA‐RCS) has been developed to enhance information security. This approach combines a hybrid chaotic permutation with a dynamic Josephus permutation and is adaptable to existing synthesis and sequencing platforms, significantly enhancing encryption strength. The system provides a security level that would require up to 3.096 × 10^106^ tests to break, ensuring robust protection against potential threats.^[^ ^205^ ^]^ Additionally, microfluidic chips used for enzymatic DNA synthesis enable the simultaneous writing and erasing of DNA, significantly improving space efficiency and offering a more effective solution for DNA storage, especially in edge computing environments.^[^ ^206^ ^]^ This system employs single‐nucleotide enzymatic DNA synthesis with a biocapping strategy, offering a cost‐effective and eco‐friendly method for data writing. In the DNA‐DISK system, 228 bits of data were successfully stored and retrieved, with a low write‐to‐read latency of just 4.4 min per bit, demonstrating its potential for efficient DNA storage.^[^ ^206^ ^]^ To address the high costs of de novo DNA synthesis, researchers have pioneered a DNA storage method that encodes digital information onto DNA molecules using DNA self‐assembly and selective enzymatic methylation technology.^[^ ^207^ ^]^ This process eliminates the need for de novo synthesis, enabling data to be written on premade nucleic acids. Using a set of 700 DNA movable types and five templates, 275 000 bits were written on an automated platform at 350 bits per reaction. This method offers a cost‐effective, scalable, and programmable approach to DNA data storage (Figure 5c).

While DNA storage technology holds promising prospects, it still faces several technical challenges, including high synthesis costs, slow sequencing speeds, and the complexity of encoding and error correction algorithms. To overcome these hurdles, interdisciplinary approaches are being explored, including new synthetic chemistry methods, nanopore sequencing technologies, and the use of cellular factories to achieve higher throughput and accuracy in reading and writing.^[^ ^208^ ^]^ Looking forward, as ongoing advancements in bioengineering and materials science continue, DNA storage is poised to be deeply integrated with large‐scale data centers, genomics analysis, and biological computing platforms. This integration aims to develop a next‐generation information storage system characterized by ultrahigh density, extremely long life, and programmable features, offering a sustainable solution to meet the escalating global data demand.^[^ ^190^ ^]^

## 6 Auxiliary Bionic Functional Structures

In the previous sections, we have conducted an in‐depth exploration of biomimetic design within electronic engineering fields, including sensing and detection systems, actuation and robotics, energy conversion and storage, and neuromorphic computing and information storage. While these discussions have primarily focused on bioinspired structures and designs directly linked to functional aspects, there has been less emphasis on overall device design. Importantly, during device operation, in addition to the core functional structures, numerous auxiliary functional structures are also involved.^[^ ^40^, ^89^, ^209^, ^210^ ^]^ Although these structures do not directly affect the device's core function, they are crucial for ensuring operational stability, durability, and environmental adaptability. Given the prevalence of these auxiliary functional structures across various device designs, and to avoid redundancy, this section is dedicated to detailing their specific roles. We will delve into the application of biomimetic design to enhance auxiliary functional structures, focusing on improving material toughness, enhancing interfacial adhesion, promoting self‐healing, and improving surface wettability. This comprehensive approach not only enriches our understanding of biomimetic design but also highlights its indispensable role in augmenting the efficiency and resilience of modern electronic systems.

### 6.1 Toughness

Enhancing material toughness is crucial for resisting vibrations, stress, or impacts, ensuring that electronic devices maintain long‐term stability in harsh environments. Nature provides numerous examples of structural toughness, which we can draw upon for biomimetic toughening designs in both flexible and rigid components of electronic devices.

**Figure 6:**
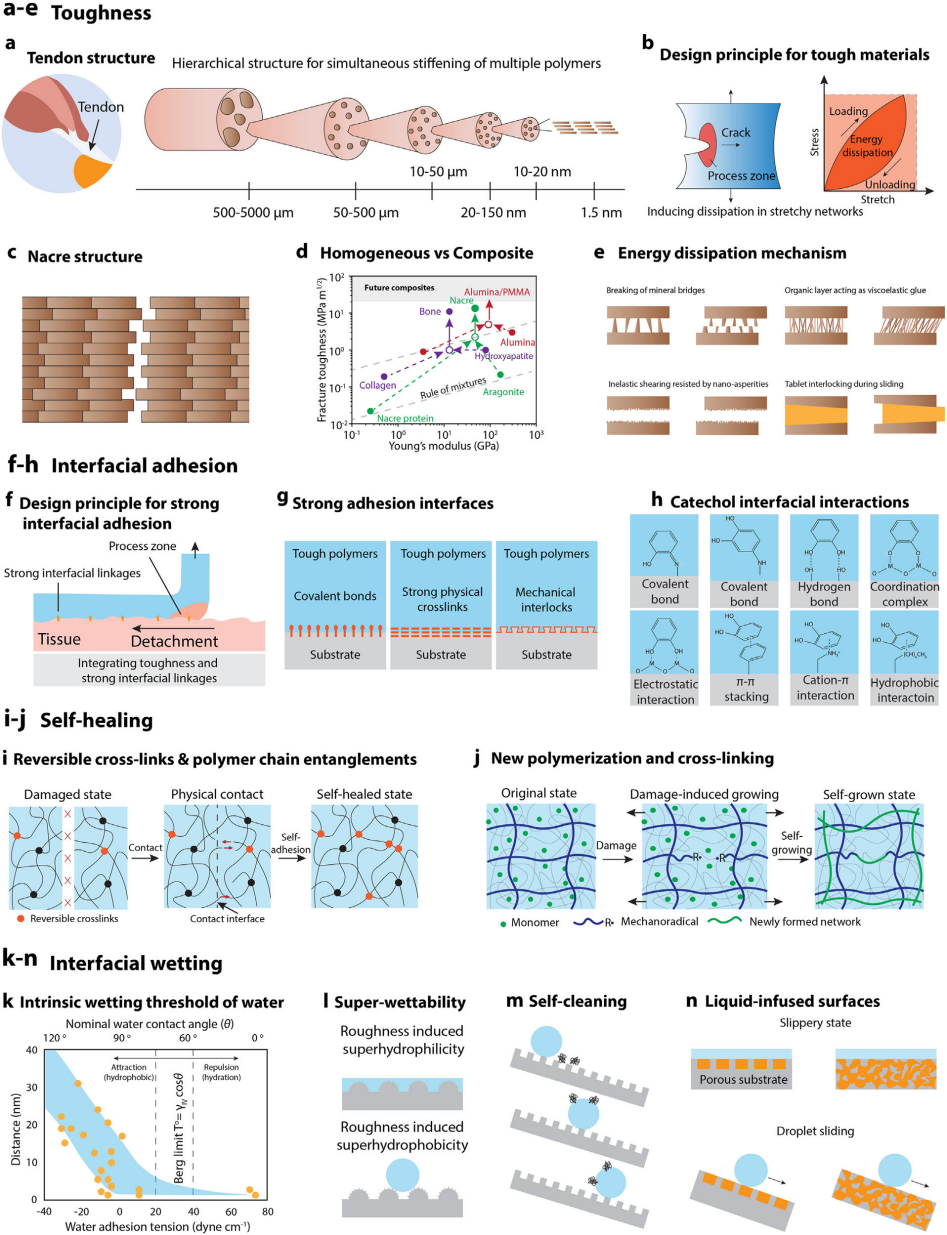
Biomimetic strategies in device auxiliary function enhancements. a–e) Biomimetic toughening designs: for soft polymers (a,b), for brittle crystals (c–e). a) Diagram illustrating the hierarchical fiber structure in tendons, contributing to their high toughness. b) Design strategies for enhancing toughness in soft materials through the integration of energy‐dissipating bonds within stretchable networks. c) Depiction of nacre's “brick‐and‐mortar” architecture, a classic example of natural toughening for brittle materials. d) Comparative analysis of the toughness in natural composites like bone and nacre, which significantly surpass that of their individual components and homogeneous combinations. Adapted with permission.^[^ ^210^ ^]^ Copyright 2015, Springer Nature. e) Detailed mechanisms of nacre's toughness, including the rupture of mineral bridges, the role of organic layers as viscoelastic connectors, inelastic shearing through nano‐asperities, and interlocking of tablets during displacement. f–h) Biomimetic adhesion designs. f) Fundamental design principle for enhancing interfacial adhesion, combining bulk material toughness with robust interfacial bonds. g) Strategies for constructing durable adhesion in soft materials, employing diverse strong interfacial connections such as covalent bonding, robust physical cross‐links, and mechanical interlocks. h) Role of catechol groups in establishing various strong interfacial bonds. i,j) Biomimetic self‐healing designs. i, Mechanisms of self‐healing via reversible cross‐linking and polymer chain entanglements at the interfaces. j) Activation of new polymerization and cross‐linking in response to damage, facilitating self‐strengthening or autonomous growth of the polymer. k–n) Biomimetic interface wettability designs for self‐cleaning. k) Quantitative analysis of “hydrophobic” and “hydrophilic” interactions based on surface force studies, highlighting the Berg limit with a contact angle around 65° indicating hydrophobic attraction. l) Enhanced effects of surface roughness on lyophilicity and lyophobicity. m) Self‐cleaning property of biomimetic superhydrophobic surfaces. n) Self‐cleaning property of biomimetic liquid‐infused surfaces.

Among flexible materials, human tendon tissue is a typical biological example. Tendons not only exhibit high tensile strength but also have excellent fracture toughness, thanks to their unique layered fiber structure (**Figure** **6**a). These structures can dissipate a significant amount of mechanical energy by hardening and fracturing collagen fibers under large deformations.^[^ ^211^ ^]^ The bionic design of tough, flexible materials mimics this toughening mechanism found in biological tissues such as cartilage, muscles, and blood vessels. In these materials, the goal is to generate a large process zone around the crack tip before the crack propagates (Figure 6b).^[^ ^212^ ^]^ For instance, by incorporating a hierarchically aligned heterogeneous structure and a gradient crosslinked network into a polyvinyl alcohol/cellulose nanofiber composite gel, a core‐sheath hydrogel (HHPC) is created that combines exceptional strength and toughness.^[^ ^213^ ^]^ This design, inspired by natural materials, provides impressive mechanical properties, including a toughness of 1031 MJ m^−3^, a strength of 55.3 MPa, and a strain of 3300%. The multilevel alignment helps prevent stress concentration at the interface between the soft and hard phases, making it an ideal substrate for flexible sensors and significantly boosting their performance.

Nacre, or abalone shell, demonstrates the ideal characteristics of natural hard materials that exhibit both strength and toughness.^[^ ^214^ ^]^ Nacre is a layered composite material made up of 95% hexagonal plate‐like hydroxyapatite or aragonite (CaCO_3_), bonded by a thin layer of organic material ≈10–50 nm thick (Figure 6c), and its toughness is three orders of magnitude higher than that of pure calcium carbonate (Figure 6d).^[^ ^210^ ^]^ The toughening mechanism in nacre primarily involves crack bridging and the mineral brick “pull‐out,” accompanied by controlled, limited sliding between the layered aragonite layers and viscoplastic energy dissipation of the organic matrix (Figure 6e). By emulating this structure, we can significantly enhance the toughness of rigid structures in synthetic bionic devices, extending their service life. For example, a nacre‐inspired pearl‐layer coating applied to the polyethylene separator in Li‐ion batteries uses lamellar slip to increase the contact area and effectively dissipate impact stress.^[^ ^215^ ^]^ This design helps preserve the separator's pore structure after external impact, ensuring consistent lithium‐ion flow during charging and discharging. It also prevents uneven lithium deposition or dendrite formation under stress. The separator, featuring porous aragonite platelets (PAPCs), exhibited superior impact resistance, with minimal voltage loss (−0.12 and −0.09 V) after impact, and better recovery performance than commercial ceramic nanoparticle‐coated separators (CNCS).

### 6.2 Interfacial Adhesion

Improving interfacial adhesion is crucial not only for ensuring stable coupling between multiple components but also plays a vital role in fields such as wearable and flexible electronics. The widespread phenomenon of adhesion in nature has sparked significant scientific interest.^[^ ^4^, ^40^, ^216^, ^217^ ^]^ For example, geckos, octopuses, tree frogs, and beetles exhibit exceptional dry/wet adhesion capabilities through the multiscale structures of their feet. Mussels, barnacles, and slugs achieve strong adhesion to various surfaces by secreting specialized biological glues, or mucus. Inspired by these natural adhesion mechanisms, researchers have developed a variety of biomimetic adhesion systems.^[^ ^211^, ^218^ ^]^ These systems typically integrate a rigid dissipative soft matrix with advanced interfacial bonding techniques.^[^ ^219^ ^]^ When external forces attempt to detach the rigid polymer from its substrate, the robust interfacial bonding forces stabilize the interfacial crack tip, allowing the polymer to form a processing zone rich in mechanical dissipation (Figure 6f). To achieve this level of tough adhesion, the inherent interfacial toughness of the biomimetic adhesion interface must at least match the intrinsic fracture energy of the rigid polymer, which is typically more than 10 joules m^−2^.^[^ ^211^ ^]^ This is generally accomplished through strategies such as covalent bonding, strong physical cross‐linking, and mechanical interlocking (Figure 6g).

Mechanical interlocking aims explicitly to achieve high‐strength adhesion between different substrates by constructing adhesives with specialized structures. For example, binders inspired by the structure of spider silk can improve interfacial adhesion through hydrogen‐bonding segments and mechanical interlocking with substrates.^[^ ^220^ ^]^ This design enables electrodes to endure substantial deformations while maintaining their integrity, ensuring stable cycling and consistent voltage output under challenging dynamic deformation tests. The binder developed for flexible lithium‐ion batteries (LIBs) contributes to a high energy density of 420 Wh L^−1^, significantly surpassing previously reported values. This innovation boosts the rate performance and output capacity at higher current densities, offering a promising material solution for high‐loading, flexible lithium‐ion batteries. Building on the design concepts of interfacial covalent bonds and strong physical cross‐linking, and inspired by the adhesion proteins found in organisms such as mussels, catechol chemistry is widely employed to achieve diverse interfacial connections between different substrates (Figure 6h).^[^ ^221^ ^]^ Upon oxidation to quinone, catechol can form covalent bonds with nucleophiles (such as amines and thiols) through Michael addition and create strong coordination complexes with metal oxides.^[^ ^221^ ^]^ Additionally, the hydroxyl groups of catechol enable electrostatic interactions with metal oxides. They can engage in cation–π interactions with positively charged functional groups via their benzene rings, as well as π–π stacking and hydrophobic interactions with hydrophobic functional groups on the substrate.^[^ ^221^ ^]^ For example, bioelectronic skin sensors inspired by the adhesion mechanisms of mussels not only demonstrate strong adhesion to dry, oily, and sweaty skin (with shear strength, tensile strength, and interfacial toughness between the hydrogel tape and wet pigskin measuring ≈1.04, 0.76 MPa, and 1021 J m^−^^2^, respectively, where dopaquinone forms chemical bonds with amino groups on the skin surface), but also offer stable and responsive electrical signal performance (≈250 ms).^[^ ^49^ ^]^ This design improves the adhesion of the interface, ensuring long‐term reliability in humid and sweaty conditions, and broadens its potential applications for real‐time bioelectric signal monitoring in complex environments.

### 6.3 Self‐Healing

Organisms possess the remarkable ability to self‐repair damage and restore their original state. This self‐healing capability offers significant advantages to artificial electronic devices by reducing damage and enhancing long‐term stability, particularly for flexible components.^[^ ^6^, ^222^ ^]^ However, biological healing processes rely on the active functions of biological cells, typically absent in artificial materials.^[^ ^223^ ^]^ In response, a common strategy for engineering materials to achieve self‐healing involves inducing the formation of new materials and/or interactions near the damaged area.^[^ ^211^ ^]^ For soft materials such as elastomers and hydrogels, this often entails creating new cross‐linking points and/or polymer chains, which is a foundational principle in designing self‐healing flexible materials (Figure 6i).

In the polymer self‐healing process, typical cross‐linking mechanisms include weak physical interactions, such as hydrogen bonds, ionic bonds, metal coordination, hydrophobic interactions, host–guest interactions, and dynamic covalent bonds.^[^ ^223^ ^]^ When two freshly formed surfaces of the damaged polymer come into contact under specific conditions, new cross‐linking points are established at the interface, thus imparting the polymer with self‐healing properties. For example, hydrogel electrolytes formed by dynamic crosslinking with borax demonstrate outstanding self‐healing and self‐adhesion properties (60.7 kPa for carbon cloth electrodes) due to the combined effects of dynamic borate ester bonds and hydrogen bonds.^[^ ^224^ ^]^ This flexibility allows supercapacitors using these hydrogel electrolytes to retain stable electrochemical performance even under continuous mechanical stress and damage, such as 180° bending, load‐bearing, and perforation. Apart from mechanisms based on weak physical interactions and dynamic covalent crosslinking, polymer self‐healing can also occur through the mutual diffusion of polymer chains and chain entanglement across the crack surface.^[^ ^223^ ^]^ Additionally, a newly reported self‐growing/self‐reinforced polymer demonstrates that chain breakage can induce the generation of mechanical free radicals, which then trigger the polymerization of monomers present in the polymer solvent (Figure 6j).^[^ ^225^ ^]^ The self‐growth or self‐reinforcement of this polymer, akin to muscle strengthening after physical training in humans, opens possibilities for constructing damage‐triggered “muscle‐building” self‐growing materials. These materials could serve as substrates for flexible sensors or as flexible electrolytes for batteries and capacitors, thereby enhancing mechanical properties. Through these innovative self‐healing strategies, artificial materials not only emulate the self‐repair abilities of organisms but also achieve higher reliability and longer service life in electronic devices. This advancement fosters the development of flexible electronic technology, potentially revolutionizing how devices are maintained and extending their functional lifespan.

### 6.4 Interfacial/Surface Wetting

Effective control of surface wettability is crucial for maintaining the stable performance of devices in humid, dusty, or other extreme environments, significantly reducing the impact of stains and corrosion.^[^ ^226^, ^227^, ^228^ ^]^ Combining multiscale structures with appropriate surface chemistry is essential for constructing interface materials with superwetting properties. The superlyophilic or superlyophobic behavior of a liquid on a rough surface is determined by its intrinsic wetting threshold (IWT), which defines the critical wettability boundary between lyophilic and lyophobic states when the liquid is deposited on an ideal (smooth and chemically uniform) solid surface (Figure 6k illustrates the IWT of water).^[^ ^229^ ^]^ A thorough understanding of the IWT of liquids is pivotal in designing superwetting surfaces. By manipulating the surface chemistry to render the solid ideally lyophilic (θ < θ_IWT_) or lyophobic (θ > θ_IWT_) and enhancing this with micro–nanostructures, superlyophilic or superlyophobic properties can be realized (Figure 6l). Over the past two decades, as many as 64 different wetting states have been identified, with superhydrophobicity and superhydrophilicity remaining primary research focuses.^[^ ^209^ ^]^ In the field of electrical engineering, drawing inspiration from the wetting properties of various biological surfaces, researchers have engineered functional interfaces, superhydrophobic, superhydrophilic, and switchable, on device surfaces to enhance device reliability and stability in complex environments. For instance, micro–nanostructure designs inspired by the lotus leaf surface greatly minimize the contact area between liquids and surfaces, providing effective waterproofing, antifouling, and self‐cleaning features (Figure 2m). This approach improves the impalement resistance, mechanical strength, and weather resistance of coatings, enabling outdoor electronic devices to perform reliably in environments with high humidity, dust, and even rain or snow, with a lifespan of 5–10 years.^[^ ^230^, ^231^ ^]^ Another biomimetic concept comes from the superslippery surface (SLIPS) of Nepenthes’ insect capsules. By infusing a low surface energy liquid into microporous or nanofiber substrates (Figure 2n), a fluid‐wettable interface can be created, suitable for microfluidic devices and anti‐icing/antifrost electronic components.^[^ ^232^ ^]^ These biomimetic wetting designs not only reduce the energy consumption and maintenance costs of electrical devices but also effectively prevent device sensitivity and lifespan from being compromised by impurities or biological contamination. In the realm of wearable and flexible electronics, by modifying the superhydrophilic/superhydrophobic interface on human skin or other flexible substrates, contact stability between the device and the skin could be enhanced, thus improving both detection accuracy and comfort.^[^ ^233^ ^]^ For example, a breathable and waterproof electronic skin (E‐skin) has been created to detect pressure and strain with distinct signals.^[^ ^234^ ^]^ The E‐skin incorporates 3D microcilia for enhanced superhydrophobicity and microscaled pores for breathability. It demonstrates a high gauge factor of 7.747 for minor strains (0%–80%) and a detection limit of 0.04%. This E‐skin can precisely differentiate various human joint movements, including bending, stretching, and pressure, and generate ternary outputs for use in logic control applications. Looking forward, the further integration of multifunctional wetting interfaces, incorporating dynamic, stimulus‐responsive, or intelligent regulation, will open up new avenues for the research and development of next‐generation high‐performance electronic devices and sensing systems.

### 6.5 Tunable‐Rigidity Structures

In addition to optical and interfacial properties, adaptive control of mechanical stiffness is a key biomimetic strategy for achieving environmental responsiveness, mechanical protection, and functional stability in electronic systems.^[^ ^235^ ^]^ In nature, various organisms adjust their rigidity to perform different mechanical tasks—muscles and tendons become stiffer during movement to enhance load‐bearing. At the same time, plant stems and echinoderms alter their stiffness in response to external stimuli. These natural systems inspire the creation of bioinspired materials with tunable rigidity, enabling them to switch between soft and rigid states in response to external signals, thereby improving resilience and adaptability. A notable example is a soft material system based on engineered variable‐occupation of water (EVO), which emulates the dual mechanical properties of both jointed and nonjointed organisms.^[^ ^236^ ^]^ By manipulating microwater distribution within the polymer matrix, the EVO gel can transition from a soft, transparent state (≈110 kPa) to a mechanically graded, opaque state (ranging from 1 MPa to 1 GPa). This reversible change in stiffness allows the material to perform both compliant deformation and rigid load‐bearing, offering dual mechanical functions within a single structure. Such water‐based stiffness modulation bridges the gap between soft and rigid materials, presenting a promising solution for adaptive substrates and intelligent mechanical systems in next‐generation electronics. Beyond material innovation, tunable‐rigidity architectures are particularly beneficial in soft robotics, wearable electronics, and adaptive biomedical systems, where devices must balance flexibility with mechanical protection.^[^ ^235^ ^]^ In flexible sensors, for instance, modulating stiffness can reduce mechanical noise or prevent excessive strain, enhancing signal accuracy and device lifespan. An example of this is a viscoelastic gelatin‐chitosan hydrogel damper, inspired by spiders' cuticular pads, that acts as an unconventional bandpass filter to eliminate dynamic mechanical noise.^[^ ^237^ ^]^ This hydrogel transitions between states depending on frequency, damping low‐frequency noise (such as walking and breathing under 30 Hz) in its rubbery state while allowing high‐frequency signals to pass in its glassy state. This adaptable filter enables high‐quality signal acquisition with minimal processing, thereby enhancing performance in bioelectronics. In soft robotic grippers, the ability to switch rigidity allows for both gentle handling and secure load holding. For instance, a shape memory polymer‐based gripper, capable of lifting objects to 6400 times its own weight, also ensures delicate interactions with fragile items like salmon eggs or newly hatched snails through soft, conformal contact.^[^ ^238^ ^]^ This illustrates how tunable stiffness can enable the gripper to adjust its force, allowing for firm lifting as well as gentle handling of delicate objects, which is vital for biomedical applications. These programmable stiffness systems highlight how biomimetic design principles can equip artificial materials with real‐time mechanical adaptability, facilitating reliable operation in complex, changing environments.

This chapter summarizes the application of auxiliary bionic functional structures in electronic devices, with a particular emphasis on their critical role in enhancing device stability, durability, and adaptability. Looking ahead, research should explore new biomimetic materials and innovative designs, focusing on dynamic, stimuli‐responsive materials that can significantly enhance the operational flexibility and environmental adaptability of devices. Additionally, the adoption of advanced manufacturing technologies, such as 3D/4D printing^[^ ^239^ ^]^ and bio‐inspired fabrication (e.g., mineralization), should be accelerated to facilitate their widespread integration across sectors, including smart cities, sustainable energy, and environmental management. By doing so, we can drive a technological revolution that leverages the unique benefits of bionic designs to meet future challenges.

## 7 Outlook

Biomimetic engineering has revitalized electrical engineering, infusing it with diverse and innovative ideas. This fresh perspective has transformed various areas, including sensor design, soft robotics, green energy collection, neuromorphic computing, and DNA storage, all of which are experiencing rapid growth. Research indicates that new bio‐inspired materials and multiscale manufacturing technologies have markedly improved the energy efficiency, responsiveness, and sustainability of electrical systems.^[^ ^3^, ^26^, ^146^ ^]^ These advancements are also paving the way for interdisciplinary developments in innovative technologies. Moreover, the seamless integration of various biomimetic strategies is propelling electrical engineering toward a new era of enhanced flexibility, agility, and efficiency.

As molecular‐level manufacturing technology and self‐assembly processes continue to evolve, the landscape of materials and structural innovation is transitioning from micro–nanoscale constructs to the domain of molecular devices. This shift not only expands the potential applications in fields such as wearable devices, visual and image sensing, and environmental and biochemical detection, but also promises to revolutionize the functionality of bionic electrical systems. For instance, inspired by the folding mechanisms and self‐organizing properties of biological proteins, researchers have engineered electrical components that exhibit self‐repair, self‐reconstruction, or programmability.^[^ ^240^ ^]^ These capabilities imbue systems with a dynamic adaptability and evolutionary potential akin to living organisms. In wearable technologies, these self‐repairing properties can dramatically increase longevity and enhance protective features, making devices more sustainable and user‐friendly. In environmental monitoring and biochemical detection, sensors equipped with programmability and adaptability can deliver real‐time data more quickly and with greater precision, enabling more responsive, effective monitoring solutions. Furthermore, integrating cutting‐edge technologies such as 4D printing and ion gels enables these systems to adapt their form and function on the fly, even under extreme conditions. This adaptability not only enhances the reliability and stability of the devices but also ensures their operational integrity in challenging environments.^[^ ^241^ ^]^ These advancements highlight the transformative potential of molecular devices, setting the stage for significant breakthroughs across sectors such as smart cities, sustainable energy, and advanced healthcare solutions.

In the realm of integrating biological and artificial intelligence, biomimetic electrical systems are set to morph into lifelike entities with autonomous learning and decision‐making capabilities.^[^ ^242^ ^]^ Leveraging neuromorphic hardware and distributed computing architectures, future circuits, and sensor networks will be able to adapt and respond to complex environments efficiently. This integration will enable them to adjust synaptic weights and enhance their multimodal perception capabilities dynamically.^[^ ^189^ ^]^ Additionally, advancements in molecular biology and synthetic biology will facilitate the seamless integration of artificial neural networks or biological computing elements with traditional electronic systems, ushering in a new era of intelligence that transcends existing technological frameworks.

In terms of interdisciplinary collaboration and social applications, biomimetic electrical engineering will move from “learning from nature” to a new stage of “coexistence with nature.” This transition involves integrating biomimetic metabolic mechanisms into electrical systems and forming symbiotic relationships with living cell systems. Such advancements pave the way for the development of highly self‐sufficient ecological electronic devices designed for challenging missions in space exploration, deep‐sea ventures, and prolonged operations in extreme environments.^[^ ^88^ ^]^ Concurrently, advancements in quantum biology and quantum computing are set to revolutionize bionic electrical systems, potentially transforming information processing and energy conversion dramatically. Essential to this evolution is the establishment of a multidisciplinary integrated platform that fosters global cooperation at the forefront of technology, propelling bionic electrical engineering to unprecedented levels.

Despite the promising potential, several significant scientific and technological hurdles must be overcome to enable the widespread adoption of biomimetic electrical systems. First, the long‐term durability and fatigue resistance of soft or hybrid bio‐inspired materials remain inadequate, as they tend to undergo mechanical degradation or interfacial delamination during repeated use.^[^ ^243^ ^]^ Second, the scalable, high‐precision fabrication of hierarchical biomimetic structures faces limitations in throughput and cost, thereby impeding their industrial application.^[^ ^244^ ^]^ Third, the field lacks clear quantitative design principles that can predict the relationships among biological structures, functional hierarchies, and device performance, thereby restricting effective optimization.^[^ ^245^ ^]^ Fourth, achieving seamless system‐level integration that combines sensing, actuation, energy conversion, and neuromorphic computation requires advancements in adaptive control logic, low‐power circuits, and machine‐learning‐driven feedback systems.^[^ ^246^ ^]^ Finally, the environmental sustainability and ethical implications of bio‐derived materials need to be thoroughly assessed through life‐cycle analysis and eco‐friendly manufacturing strategies. Overcoming these challenges will improve the stability, reproducibility, and scalability of biomimetic electrical systems, facilitating their evolution from conceptual prototypes to reliable, intelligent, and environmentally sustainable technologies.

In summary, the application prospects of biomimetic engineering in electrical engineering are expansive, continually redefining the boundaries of traditional engineering practices. By harnessing multidisciplinary expertise and addressing fundamental challenges in materials, processes, integration, and algorithms in practical application contexts, biomimetic electrical engineering is poised to deliver breakthrough innovations. These advancements will not only yield more original solutions but also provide robust support for a sustainable, intelligent society.

## Conflict of Interest

The authors declare no conflict of interest.

## Author Contributions

M.L. and A.M. contributed equally to this work. M.L. and A.M. contributed to literature searching, writing, and editing of this manuscript. A.M. drew figures for the article and contributed to the discussion of content. Q.G., Y.X., C.L., G.L., and E.S. contributed to the discussion, writing, and reviewing/editing of the manuscript before submission. All authors discussed and contributed to the writing of the manuscript.

## References

[1] S. Ullah , G. Xie , J. R. Gong , Microelectron. Eng. 2024, 293, 112230.

[2] D. Stuart‐Fox , L. Ng , M. A. Elgar , K. Hölttä‐Otto , G. E. Schröder‐Turk , N. H. Voelcker , G. S. Watson , Nat. Rev. Mater. 2023, 8, 565.

[3] W. Li , Q. Guan , M. Li , E. Saiz , X. Hou , Prog. Polym. Sci. 2023, 140, 101665.

[4] M. Li , A. Mao , Q. Guan , E. Saiz , Chem. Soc. Rev. 2024, 53, 8240. 38982929 10.1039/d3cs00764b

[5] T. Ozaki , N. Ohta , M. Fujiyoshi , Nat. Commun. 2025, 16, 1196. 39885178 10.1038/s41467-025-56596-1 PMC11782484

[6] Y. Zhou , L. Li , Z. Han , Q. Li , J. He , Q. Wang , Chem. Rev. 2022, 123, 558. 36260027 10.1021/acs.chemrev.2c00231

[7] B. Nie , S. Liu , Q. Qu , Y. Zhang , M. Zhao , J. Liu , Acta Biomater. 2022, 139, 280. 34157454 10.1016/j.actbio.2021.06.018

[8] B. Wang , C. Wang , Y. Li , J. Jin , X. Lin , C. Shi , Energy Environ. Sci. 2025, 18, 3432.

[9] X. Hou , L. Xin , Y. Fu , Z. Na , G. Gao , Y. Liu , Q. Xu , P. Zhao , G. Yan , Y. Su , Nano Energy 2023, 118, 109034.

[10] K. Mahato , T. Saha , S. Ding , S. S. Sandhu , A.‐Y. Chang , J. Wang , Nat. Electron. 2024, 7, 735.

[11] X. Wu , S. Shi , J. Jiang , D. Lin , J. Song , Z. Wang , W. Huang , Adv. Mater. 2025, 37, 2419159. 10.1002/adma.202419159 39945055

[12] B. Ding , Y. Ding , J. Peng , J. Romano‐deGea , L. E. Frederiksen , H. Kanda , O. A. Syzgantseva , M. A. Syzgantseva , J.‐N. Audinot , J. Bour , Nature 2024, 628, 299. 38438066 10.1038/s41586-024-07228-z PMC11006611

[13] A. Kobayashi , S.‐y. Takizawa , M. Hirahara , Coord. Chem. Rev. 2022, 467, 214624.

[14] Y. H. Hong , Y.‐M. Lee , W. Nam , S. Fukuzumi , J. Am. Chem. Soc. 2022, 144, 695. 34990144 10.1021/jacs.1c11707

[15] Z. Wang , N. M. Freris , X. S. R. Wei , Device 2024, 3, 100646.

[16] J. Sun , S. Zhang , J. Deng , J. Li , D. Wang , J. Liu , Y. Liu , SmartBot 2025, 1, 12005.

[17] S. Zhuo , Z. Zhao , Z. Xie , Y. Hao , Y. Xu , T. Zhao , H. Li , E. M. Knubben , L. Wen , L. Jiang , Sci. Adv. 2020, 6, aax1464. 10.1126/sciadv.aax1464 PMC6994219 32064332

[18] X. Dong , C. Wang , H. Song , J. Shao , G. Lan , J. Zhang , X. Li , M. Li , Biomimetics 2024, 9, 585. 39451793 10.3390/biomimetics9100585 PMC11505285

[19] J. Yan , J. P. Armstrong , F. Scarpa , A. W. Perriman , Adv. Mater. 2024, 36, 2403937. 10.1002/adma.202403937 39087845

[20] H. Jeong , S. Han , S.‐O. Park , T. R. Kim , J. Bae , T. Jang , Y. Cho , S. Seo , H.‐J. Jeong , S. Park , Nat. Electron. 2025, 8, 168.

[21] T. Wang , J. Meng , X. Zhou , Y. Liu , Z. He , Q. Han , Q. Li , J. Yu , Z. Li , Y. Liu , Nat. Commun. 2022, 13, 7432. 36460675 10.1038/s41467-022-35160-1 PMC9718838

[22] H.‐K. Kim , Y.‐G. Cha , J.‐M. Kwon , S.‐I. Bae , K. Kim , K.‐W. Jang , Y.‐J. Jo , M. H. Kim , K.‐H. Jeong , Sci. Adv. 2025, 11, ads3389. 10.1126/sciadv.ads3389 PMC11691699 39742496

[23] T.‐C. Tang , B. An , Y. Huang , S. Vasikaran , Y. Wang , X. Jiang , T. K. Lu , C. Zhong , Nat. Rev. Mater. 2021, 6, 332.

[24] D. Pan , J. Hu , B. Wang , X. Xia , Y. Cheng , C. H. Wang , Y. Lu , Adv. Sci. 2024, 11, 2303264. 10.1002/advs.202303264 PMC10837381 38044298

[25] X. Fu , W. Cheng , G. Wan , Z. Yang , B. C. Tee , Chem. Rev. 2024, 124, 9899. 39198214 10.1021/acs.chemrev.4c00049 PMC11397144

[26] C. Hegde , J. Su , J. M. R. Tan , K. He , X. Chen , S. Magdassi , ACS Nano 2023, 17, 15277. 37530475 10.1021/acsnano.3c04089 PMC10448757

[27] A. B. Grommet , M. Feller , R. Klajn , Nat. Nanotechnol. 2020, 15, 256. 32303705 10.1038/s41565-020-0652-2

[28] C. Cai , Q. Hu , Q. Li , W. Peng , Y. Sun , F. Tang , Y. Liu , J. Wang , B. Luo , X. Li , Chem. Eng. J. 2025, 522, 168250.

[29] X. Gou , C. Liao , Y. Zhang , P. Li , S. Lang , C. Zhang , S. Hu , N. Yu , C. Li , J. Yang , Composites, Part B 2026, 309, 113043.

[30] P. Xu , X. Wang , S. Wang , T. Chen , J. Liu , J. Zheng , W. Li , M. Xu , J. Tao , G. Xie , Research 2021, 2021, 9864967. 38617376 10.34133/2021/9864967 PMC11014677

[31] J. Liu , P. Xu , J. Zheng , X. Liu , X. Wang , S. Wang , T. Guan , G. Xie , M. Xu , Nano Energy 2022, 101, 107633.

[32] W. Wang , Y. Liu , M. Ding , T. Xia , Q. Gong , X. Zeng , Z. Cai , Y. Hu , Nano Energy 2023, 116, 108832.

[33] J.‐H. Lee , Y.‐N. Kim , J. Lee , J. Jeon , J.‐Y. Bae , J.‐Y. Lee , K.‐S. Kim , M. Chae , H. Park , J.‐h. Kim , Sci. Adv. 2024, 10, ads9258. 10.1126/sciadv.ads9258 PMC11661431 39705343

[34] M. Asadnia , A. G. P. Kottapalli , K. D. Karavitaki , M. E. Warkiani , J. Miao , D. P. Corey , M. Triantafyllou , Sci. Rep. 2016, 6, 32955. 27622466 10.1038/srep32955 PMC5020657

[35] X. Hu , Y. Jiang , Z. Ma , Y. Xu , D. Zhang , Sensors 2019, 19, 5384. 31817605 10.3390/s19245384 PMC6960935

[36] Z.‐Y. Hu , Y.‐L. Zhang , C. Pan , J.‐Y. Dou , Z.‐Z. Li , Z.‐N. Tian , J.‐W. Mao , Q.‐D. Chen , H.‐B. Sun , Nat. Commun. 2022, 13, 5634. 36163128 10.1038/s41467-022-33072-8 PMC9513083

[37] Y. Zhou , Z. Sun , Y. Ding , Z. Yuan , X. Qiu , Y. B. Cao , Z. A. Wan , Z. Long , S. Poddar , S. Kumar , Sci. Rob. 2024, 9, adi8666. 10.1126/scirobotics.adi8666 38748782

[38] M. Li , Q. Guan , C. Li , E. Saiz , Device 2023, 1, 100007.

[39] M. Yuan , Y. Long , T. Liu , J. Liu , S. Qiu , T. Lin , F. Xu , Y. Fang , Mater. Today 2024, 75, 166.

[40] X. Wei , Y. Wang , Y. Liu , K. Ji , K. Li , J. Wang , Z. Gu , Matter 2024, 7, 826.

[41] T. Someya , M. Amagai , Nat. Biotechnol. 2019, 37, 382. 30940942 10.1038/s41587-019-0079-1

[42] B. Ying , X. Liu , Iscience 2021, 103174, 24,. 34755087 10.1016/j.isci.2021.103174 PMC8564057

[43] Z. W. K. Low , Z. Li , C. Owh , P. L. Chee , E. Ye , D. Kai , D. P. Yang , X. J. Loh , Small 2019, 15, 1805453. 10.1002/smll.201805453 30690897

[44] C. Wang , C. Wang , Z. Huang , S. Xu , Adv. Mater. 2018, 30, 1801368. 10.1002/adma.201801368 30073715

[45] X. Chen , C. Wang , W. Wei , Y. Liu , S. S. Ge , L. Zhou , H. Kong , npj Flexible Electron. 2025, 9, 101.

[46] Q. Huang , Y. Jiang , Z. Duan , Y. Wu , Z. Yuan , J. Guo , M. Zhang , H. Tai , Nano Energy 2024, 126, 109689.

[47] S. Buoso , B. T. Dickinson , R. Palacios , Bioinspiration Biomimetics 2017, 13, 016013. 29283112 10.1088/1748-3190/aa9a7b

[48] Q. Xin , J. Zhang , Z. Han , H. Zhao , T. Hou , Y. Liu , S. Niu , Q. Han , Z. Mu , B. Li , Adv. Mater. Technol. 2023, 8, 2200756.

[49] M. Li , W. Li , Q. Guan , J. Lv , Z. Wang , L. Ding , C. Li , E. Saiz , X. Hou , Device 2023, 100006.

[50] Y. Hao , Q. Yan , H. Liu , X. He , P. Zhang , X. Qin , R. Wang , J. Sun , L. Wang , Y. Cheng , Adv. Funct. Mater. 2023, 33, 2303881.

[51] Y. Ni , X. Zang , Y. Yang , Z. Gong , H. Li , J. Chen , C. Wu , J. Huang , Y. Lai , Adv. Funct. Mater. 2024, 34, 2402853.

[52] Z. Zhang , L. Wang , C. Lee , Adv. Sensor Res. 2023, 2, 2200072.

[53] A. G. Jaramillo , M. E. Benalcazar , in 2017 IEEE Second Ecuador Technical Chapters Meeting (ETCM), IEEE, New York 2017, p. 1.

[54] L. Zhao , S. Jia , C. Fang , B. Qin , Y. Hu , X. Yang , Chem. Eng. J. 2025, 503, 158637.

[55] G.‐T. Xiang , N. Chen , B. Lu , J.‐L. Xu , R. D. Rodriguez , E. Sheremet , Y.‐D. Hu , J.‐J. Chen , Nano Energy 2023, 118, 108936.

[56] D. Berson , Neuron 2021, 109, 1418. 33957069 10.1016/j.neuron.2021.04.018

[57] Z. Long , Y. Zhou , Y. Ding , X. Qiu , S. Poddar , Z. Fan , Nat. Rev. Mater. 2025, 10, 128.

[58] L. Gu , S. Poddar , Y. Lin , Z. Long , D. Zhang , Q. Zhang , L. Shu , X. Qiu , M. Kam , A. Javey , Nature 2020, 581, 278. 32433619 10.1038/s41586-020-2285-x

[59] B. Dai , L. Zhang , C. Zhao , H. Bachman , R. Becker , J. Mai , Z. Jiao , W. Li , L. Zheng , X. Wan , Nat. Commun. 2021, 12, 6458. 34753909 10.1038/s41467-021-26606-z PMC8578215

[60] H. Jiang , C. C. Tsoi , L. Sun , W. Yu , H. Fan , M. Ma , Y. Jia , X. Zhang , Adv. Devices Instrument. 2024, 5, 0034.

[61] Z. Xuan , J. Li , Q. Liu , F. Yi , S. Wang , W. Lu , The Innovation 2021, 2, 100081. 10.1016/j.xinn.2021.100081 PMC8454771 34557736

[62] F. Meng , B. Ju , Z. Wang , R. Han , Y. Zhang , S. Zhang , P. Wu , B. Tang , J. Am. Chem. Soc. 2022, 144, 7610. 35446030 10.1021/jacs.2c02894

[63] Y. Shang , C. Huang , Z. Li , X. Du , Adv. Funct. Mater. 2025, 35, 2412703.

[64] D. Kou , S. Zhang , W. Ma , Adv. Opt. Mater. 2024, 12, 2400192.

[65] T. Liu , R. Meng , J. Ren , Y. Han , X. Li , S. Zhang , S. Wu , Chem. Eng. J. 2024, 489, 151277.

[66] S. H. Choi , D. Kim , Y. Lee , S. Hong , J. Lee , J. Jeong , J. Su , H. Lim , S. H. Ko , Nat. Rev. Bioeng. 2025, 3, 579.

[67] H. Kim , J. Choi , K. K. Kim , P. Won , S. Hong , S. H. Ko , Nat. Commun. 2021, 12, 4658. 34376680 10.1038/s41467-021-24916-w PMC8355336

[68] J. Lee , H. Sul , Y. Jung , H. Kim , S. Han , J. Choi , J. Shin , D. Kim , J. Jung , S. Hong , Adv. Funct. Mater. 2020, 30, 2003328.

[69] E. Amalfitano , K. Pardee , Nat. Chem. Biol. 2022, 18, 356. 35177838 10.1038/s41589-021-00963-8

[70] M. A. Boyd , W. Thavarajah , J. B. Lucks , N. P. Kamat , Sci. Adv. 2023, 9, add6605. 10.1126/sciadv.add6605 PMC9812392 36598992

[71] S. Paul , S. Lakshmi , D. Akula , A. Thekkangil , Harnessing Microbial Potential for Multifarious Applications, Springer, Singapore 2024, p. 439.

[72] X. Wan , B. Saltepe , L. Yu , B. Wang , Microb. Biotechnol. 2021, 14, 2334. 33960658 10.1111/1751-7915.13820 PMC8601174

[73] J. Capin , E. Chabert , A. Zuñiga , J. Bonnet , Microb. Biotechnol. 2024, 17, 70047. 10.1111/1751-7915.70047 PMC11568237 39548716

[74] X. Wu , R. Wang , N. Kwon , H. Ma , J. Yoon , Chem. Soc. Rev. 2022, 51, 450. 34951429 10.1039/d1cs00543j

[75] Y. Hu , Y. Cui , Z. Zhang , X. Zhang , X. Ma , Z. Qiao , F. Zheng , F. Feng , W. Liu , L. Han , Anal. Chem. 2024, 96, 11205. 38967035 10.1021/acs.analchem.4c00455

[76] D. Li , W. He , X. Lin , X. Cui , S. Nagl , A. R. Wu , R. T. Kwok , R. Wu , B. Z. Tang , Aggregate 2023, 4, 384.

[77] L. Liu , N. Na , J. Yu , W. Zhao , Z. Wang , Y. Zhu , C. Hu , Adv. Sci. 2024, 11, 2305639. 10.1002/advs.202305639 PMC10870059 38095453

[78] J. Yang , X. Hu , L. Feng , Z. Liu , A. Murtazt , W. Qin , M. Zhou , J. Liu , Y. Bi , J. Qian , ACS Sens. 2024, 9, 2925. 38836922 10.1021/acssensors.4c00050

[79] Z. Liu , Q. Liu , W. Xu , L. Wang , Z. Zhou , Rob. Comput.‐Integr. Manuf. 2022, 77, 102360.

[80] J. Bang , S. H. Choi , K. R. Pyun , Y. Jung , S. Hong , D. Kim , Y. Lee , D. Won , S. Jeong , W. Shin , Nat. Rev. Electr. Eng. 2024, 1, 597.

[81] I. Must , E. Sinibaldi , B. Mazzolai , Nat. Commun. 2019, 10, 344. 30664648 10.1038/s41467-018-08173-y PMC6341089

[82] X. Ding , J. Chen , Z. Liu , Z. Liu , Y. Zhu , Adv. Funct. Mater. 2025, 18322.

[83] Z. Xie , F. Yuan , J. Liu , L. Tian , B. Chen , Z. Fu , S. Mao , T. Jin , Y. Wang , X. He , Sci. Rob. 2023, 8, adh7852. 10.1126/scirobotics.adh7852 38019929

[84] S. Leanza , J. Lu‐Yang , B. Kaczmarski , S. Wu , E. Kuhl , R. R. Zhao , Adv. Funct. Mater. 2024, 34, 2400396.

[85] J. Tirado , C. D. Do , J. Moisson de Vaux , J. Jørgensen , A. Rafsanjani , Adv. Sci. 2024, 11, 2400012. 10.1002/advs.202400012 PMC11187925 38622890

[86] P. Li , Y. Zhang , G. Zhang , D. Zhou , L. Li , Research 2021, 2021, 9843859. 34778791 10.34133/2021/9843859 PMC8557356

[87] C. Xu , Y. Cao , J. Zhao , Y. Cao , Y. Huang , Y. Lin , D. Wang , Z. Zhang , H. Jiang , Nat. Commun. 2025, 16, 6813. 40707471 10.1038/s41467-025-62182-2 PMC12289928

[88] G. Li , T.‐W. Wong , B. Shih , C. Guo , L. Wang , J. Liu , T. Wang , X. Liu , J. Yan , B. Wu , Nat. Commun. 2023, 14, 7097. 37925504 10.1038/s41467-023-42882-3 PMC10625581

[89] Y. Roh , Y. Lee , D. Lim , D. Gong , S. Hwang , M. Kang , D. Kim , J. Cho , G. Kwon , D. Kang , Adv. Funct. Mater. 2024, 34, 2306079.

[90] M. Ilami , H. Bagheri , R. Ahmed , E. O. Skowronek , H. Marvi , Adv. Mater. 2021, 33, 2003139. 10.1002/adma.202003139 33346386

[91] P. Won , S. H. Ko , C. Majidi , A. W. Feinberg , V. A. Webster‐Wood , Actuators 2020, 9, 96.

[92] Y. Jung , K. Kwon , J. Lee , S. H. Ko , Nat. Commun. 2024, 15, 3510. 38664373 10.1038/s41467-024-47639-0 PMC11045848

[93] H. Kim , S.‐k. Ahn , D. M. Mackie , J. Kwon , S. H. Kim , C. Choi , Y. H. Moon , H. B. Lee , S. H. Ko , Mater. Today 2020, 41, 243.

[94] J. Lee , Y. Yoon , H. Park , J. Choi , Y. Jung , S. H. Ko , W.‐H. Yeo , Adv. Intell. Syst. 2022, 4, 2100271.

[95] H. Kim , H. Lee , I. Ha , J. Jung , P. Won , H. Cho , J. Yeo , S. Hong , S. Han , J. Kwon , Adv. Funct. Mater. 2018, 28, 1801847.

[96] Y. Yoon , H. Park , J. Lee , J. Choi , Y. Jung , S. Han , I. Ha , S. H. Ko , Chem. Eng. J. 2023, 451, 138794.

[97] J. Ko , C. Kim , D. Kim , Y. Song , S. Lee , B. Yeom , J. Huh , S. Han , D. Kang , J.‐S. Koh , Sci. Rob. 2022, 7, abo6463. 10.1126/scirobotics.abo6463 36288271

[98] J. Ko , D. Kim , Y. Song , S. Lee , M. Kwon , S. Han , D. Kang , Y. Kim , J. Huh , J.‐S. Koh , ACS Nano 2020, 14, 11906. 32885947 10.1021/acsnano.0c04899

[99] H. Yang , M. Xu , W. Li , S. Zhang , IEEE Trans. Ind. Electron. 2018, 66, 6108.

[100] M. S. Kim , J. K. Heo , H. Rodrigue , H. T. Lee , S. Pané , M. W. Han , S. H. Ahn , Adv. Mater. 2023, 35, 2208517. 10.1002/adma.202208517 37074738

[101] H. Zhang , H. Shea , Adv. Funct. Mater. 2025, 35, 2415099.

[102] A. Lendlein , O. E. Gould , Nat. Rev. Mater. 2019, 4, 116.

[103] B. Aksoy , H. Shea , Adv. Funct. Mater. 2020, 30, 2001597.

[104] K. Ly , N. Kellaris , D. McMorris , B. K. Johnson , E. Acome , V. Sundaram , M. Naris , J. S. Humbert , M. E. Rentschler , C. Keplinger , Soft Rob. 2021, 8, 673. 10.1089/soro.2020.0048 33001742

[105] R. Wang , C. Zhang , W. Tan , J. Yang , D. Lin , L. Liu , Soft Rob. 2023, 10, 119. 10.1089/soro.2021.0104 35482290

[106] L. Yang , H. Wang , Acta Biomater. 2024, 185, 24. 39025393 10.1016/j.actbio.2024.07.016

[107] B. Mazzolai , A. Mondini , E. Del Dottore , L. Margheri , F. Carpi , K. Suzumori , M. Cianchetti , T. Speck , S. K. Smoukov , I. Burgert , Multifunct. Mater. 2022, 5, 032001.

[108] Z. Zhang , Y. Long , G. Chen , Q. Wu , H. Wang , H. Jiang , Sci. Adv. 2023, 9, adg1203. 10.1126/sciadv.adg1203 PMC10096572 37043577

[109] N. W. Bartlett , M. T. Tolley , J. T. Overvelde , J. C. Weaver , B. Mosadegh , K. Bertoldi , G. M. Whitesides , R. J. Wood , Science 2015, 349, 161. 26160940 10.1126/science.aab0129

[110] D. Drotman , S. Jadhav , D. Sharp , C. Chan , M. T. Tolley , Sci. Rob. 2021, 6, aay2627. 10.1126/scirobotics.aay2627 34043527

[111] C. A. Aubin , R. H. Heisser , O. Peretz , J. Timko , J. Lo , E. F. Helbling , S. Sobhani , A. D. Gat , R. F. Shepherd , Science 2023, 381, 1212. 37708265 10.1126/science.adg5067

[112] Y. Zhang , X. Zhu , H. Chen , R. Wang , S. Qiu , H. Zhang , H. Li , Z. Li , R. Wang , F. Zhang , Addit. Manuf. 2024, 94, 104513.

[113] Y. Alapan , A. C. Karacakol , S. N. Guzelhan , I. Isik , M. Sitti , Sci. Adv. 2020, 6, abc6414. 10.1126/sciadv.abc6414 PMC7500935 32948594

[114] Z. Li , N. V. Myung , Y. Yin , Sci. Rob. 2021, 6, abi4523. 10.1126/scirobotics.abi4523 34851711

[115] S. Wu , Y. Hong , Y. Zhao , J. Yin , Y. Zhu , Sci. Adv. 2023, 9, adf8014. 10.1126/sciadv.adf8014 PMC10032605 36947625

[116] G. Cai , J.‐H. Ciou , Y. Liu , Y. Jiang , P. S. Lee , Sci. Adv. 2019, 5, aaw7956. 10.1126/sciadv.aaw7956 PMC6625817 31309158

[117] Y. Cui , D. Li , C. Gong , C. Chang , ACS Nano 2021, 15, 13712. 34396782 10.1021/acsnano.1c05019

[118] Y. Zhang , W. Zhang , J. Yang , W. Pu , Soft Rob. 2022, 9, 531. 10.1089/soro.2021.0009 34115957

[119] Y. Guo , L. Liu , Y. Liu , J. Leng , Adv. Intell. Syst. 2021, 3, 2000282.

[120] D. Wang , B. Zhao , X. Li , L. Dong , M. Zhang , J. Zou , G. Gu , Nat. Commun. 2023, 14, 5067. 37604806 10.1038/s41467-023-40626-x PMC10442442

[121] G. Mengaldo , F. Renda , S. L. Brunton , M. Bächer , M. Calisti , C. Duriez , G. S. Chirikjian , C. Laschi , Nat. Rev. Phys. 2022, 4, 595.

[122] D. Kim , S.‐H. Kim , T. Kim , B. B. Kang , M. Lee , W. Park , S. Ku , D. Kim , J. Kwon , H. Lee , PLoS One 2021, 16, 0246102. 10.1371/journal.pone.0246102 PMC7891779 33600496

[123] K. Chin , T. Hellebrekers , C. Majidi , Adv. Intell. Syst. 2020, 2, 1900171.

[124] B. Shih , D. Shah , J. Li , T. G. Thuruthel , Y.‐L. Park , F. Iida , Z. Bao , R. Kramer‐Bottiglio , M. T. Tolley , Sci. Rob. 2020, 5, aaz9239. 10.1126/scirobotics.aaz9239 33022628

[125] T. Sun , B. Feng , J. Huo , Y. Xiao , W. Wang , J. Peng , Z. Li , C. Du , W. Wang , G. Zou , Nano‐Micro Lett. 2024, 16, 14. 10.1007/s40820-023-01235-x PMC10643743 37955844

[126] S. Gong , W. Li , J. Wu , B. Feng , Z. Yi , X. Guo , W. Zhang , L. Shao , Adv. Sci. 2024, 11, 2308835. 10.1002/advs.202308835 PMC11200028 38647364

[127] C.‐H. Li , C. Wang , C. Keplinger , J.‐L. Zuo , L. Jin , Y. Sun , P. Zheng , Y. Cao , F. Lissel , C. Linder , Nat. Chem. 2016, 8, 618. 27219708 10.1038/nchem.2492

[128] S. M. Mirvakili , I. W. Hunter , Adv. Mater. 2018, 30, 1704407. 10.1002/adma.201704407 29250838

[129] X. Ren , Y. Morimoto , S. Takeuchi , Sci. Rob. 2025, 10, adr5512. 10.1126/scirobotics.adr5512 39937887

[130] X. Leng , X. Hu , W. Zhao , B. An , X. Zhou , Z. Liu , Adv. Intell. Syst. 2021, 3, 2000185.

[131] Y. Wan , Y. Zhong , A. Ma , L. Zhang , IEEE Trans. Cybern. 2022, 53, 2658. 10.1109/TCYB.2022.3170580 35604984

[132] W. Husheng , L. Hao , X. Renbin , J. Syst. Eng. Electron. 2021, 32, 1180.

[133] H. Ming , C. Haotian , H. Wei , D. Cheng , D. Haibin , Acta Aeronaut. Astronaut. Sin. 2024, 45, 029946.

[134] X. Hai , H. Qiu , C. Wen , Q. Feng , IEEE Trans. Intell. Transport. Syst. 2023, 24, 14706.

[135] C. Bai , P. Yan , H. Piao , W. Pan , J. Guo , IEEE Trans. Cybern. 2023, 54, 462. 37028361 10.1109/TCYB.2023.3246985

[136] J. Tang , H. Duan , S. Lao , Artif. Intell. Rev. 2023, 56, 4295.

[137] L. Xu , X. Cao , W. Du , Y. Li , Knowledge‐Based Syst. 2023, 260, 110164.

[138] J. Wubben , D. Hernández , J. M. Cecilia , B. Imberón , C. T. Calafate , J.‐C. Cano , P. Manzoni , C. K. Toh , IEEE Trans. Intell. Transport. Syst. 2023, 24, 4836.

[139] C. Gao , J. Ma , T. Li , Y. Shen , Complex Intell. Syst. 2023, 9, 1929.

[140] Y. Chen , S. Xu , Z. Ren , P. Chirarattananon , IEEE Trans. Rob. 2021, 37, 1752.

[141] A. Goldthau , S. Tagliapietra , Nature 2022, 612, 627. 36526908 10.1038/d41586-022-04467-w

[142] D. Welsby , J. Price , S. Pye , P. Ekins , Nature 2021, 597, 230. 34497394 10.1038/s41586-021-03821-8

[143] J. Lv , J. Xie , A. G. A. Mohamed , X. Zhang , Y. Feng , L. Jiao , E. Zhou , D. Yuan , Y. Wang , Nat. Rev. Chem. 2023, 7, 91. 37117911 10.1038/s41570-022-00448-9

[144] K. Catania , Science 2014, 346, 1231. 25477462 10.1126/science.1260807

[145] J. V. Boas , V. B. Oliveira , M. Simões , A. M. Pinto , J. Environ. Manag. 2022, 307, 114525. 10.1016/j.jenvman.2022.114525 35091241

[146] B. Zhang , W. Xu , L. Peng , Y. Li , W. Zhang , Z. Wang , Nat. Rev. Electr. Eng. 2024, 1, 218.

[147] M. Li , C. Li , B. R. Blackman , E. Saiz , Matter 2021, 4, 3400.

[148] Z. Liu , X. Chen , Z. L. Wang , Adv. Mater. 2024, 37, 2409440.

[149] T. Cheng , J. Shao , Z. L. T Wang , Nat. Rev. Methods Primers 2023, 3, 39.

[150] L. Wang , Y. Song , W. Xu , W. Li , Y. Jin , S. Gao , S. Yang , C. Wu , S. Wang , Z. Wang , EcoMat 2021, 3, 12116.

[151] W. Xu , X. Zhou , C. Hao , H. Zheng , Y. Liu , X. Yan , Z. Yang , M. Leung , X. C. Zeng , R. X. Xu , Natl. Sci. Rev. 2019, 6, 540. 34691903 10.1093/nsr/nwz025 PMC8291521

[152] Y. Song , W. Xu , Y. Liu , H. Zheng , M. Cui , Y. Zhou , B. Zhang , X. Yan , L. Wang , P. Li , Innovation 2022, 3, 100301. 36051817 10.1016/j.xinn.2022.100301 PMC9425077

[153] S. Zhang , M. Chi , J. Mo , T. Liu , Y. Liu , Q. Fu , J. Wang , B. Luo , Y. Qin , S. Wang , Nat. Commun. 2022, 13, 4168. 35851036 10.1038/s41467-022-31987-w PMC9293931

[154] C. Ballif , F.‐J. Haug , M. Boccard , P. J. Verlinden , G. Hahn , Nat. Rev. Mater. 2022, 7, 597.

[155] S. Mathew , A. Yella , P. Gao , R. Humphry‐Baker , B. F. Curchod , N. Ashari‐Astani , I. Tavernelli , U. Rothlisberger , M. K. Nazeeruddin , M. Grätzel , Nat. Chem. 2014, 6, 242. 24557140 10.1038/nchem.1861

[156] N. Soudi , S. Nanayakkara , N. M. Jahed , S. Naahidi , Sol. Energy 2020, 208, 31.

[157] R. Schmager , B. Fritz , R. Hünig , K. Ding , U. Lemmer , B. S. Richards , G. Gomard , U. W. Paetzold , ACS Photonics 2017, 4, 2687.

[158] R. H. Siddique , Y. J. Donie , G. Gomard , S. Yalamanchili , T. Merdzhanova , U. Lemmer , H. Hölscher , Sci. Adv. 2017, 3, 1700232. 10.1126/sciadv.1700232 PMC5648565 29057320

[159] J. Sun , X. Wang , J. Wu , C. Jiang , J. Shen , M. A. Cooper , X. Zheng , Y. Liu , Z. Yang , D. Wu , Sci. Rep. 2018, 8, 5438. 29615712 10.1038/s41598-018-23771-y PMC5883013

[160] R. Hünig , A. Mertens , M. Stephan , A. Schulz , B. Richter , M. Hetterich , M. Powalla , U. Lemmer , A. Colsmann , G. Gomard , Adv. Opt. Mater. 2016, 4, 1487.

[161] X. Qian , Y. Zhao , Y. Alsaid , X. Wang , M. Hua , T. Galy , H. Gopalakrishna , Y. Yang , J. Cui , N. Liu , Nat. Nanotechnol. 2019, 14, 1048. 31686005 10.1038/s41565-019-0562-3

[162] X.‐L. Shi , J. Zou , Z.‐G. Chen , Chem. Rev. 2020, 120, 7399. 32614171 10.1021/acs.chemrev.0c00026

[163] L. Li , S. Feng , Y. Bai , X. Yang , M. Liu , M. Hao , S. Wang , Y. Wu , F. Sun , Z. Liu , Nat. Commun. 2022, 13, 1043. 35210414 10.1038/s41467-022-28689-8 PMC8873497

[164] W. Ren , Y. Sun , D. Zhao , A. Aili , S. Zhang , C. Shi , J. Zhang , H. Geng , J. Zhang , L. Zhang , Sci. Adv. 2021, 7, abe0586. 10.1126/sciadv.abe0586 PMC7875524 33568483

[165] J.‐H. Meng , Y. Liu , X.‐H. Zhu , Z.‐J. Yang , K. Zhang , G. Lu , Energy Convers. Manage. 2022, 273, 116404.

[166] T. Li , X. Zhang , S. D. Lacey , R. Mi , X. Zhao , F. Jiang , J. Song , Z. Liu , G. Chen , J. Dai , Nat. Mater. 2019, 18, 608. 30911121 10.1038/s41563-019-0315-6

[167] N. N. Shi , C.‐C. Tsai , F. Camino , G. D. Bernard , N. Yu , R. Wehner , Science 2015, 349, 298. 26089358 10.1126/science.aab3564

[168] M. Lee , G. Kim , Y. Jung , K. R. Pyun , J. Lee , B.‐W. Kim , S. H. Ko , Light: Sci. Appl. 2023, 12, 134. 37264035 10.1038/s41377-023-01119-0 PMC10235094

[169] H. Zhang , K. C. Ly , X. Liu , Z. Chen , M. Yan , Z. Wu , X. Wang , Y. Zheng , H. Zhou , T. Fan , Proc. Natl. Acad. Sci. USA 2020, 117, 14657. 32541048 10.1073/pnas.2001802117 PMC7334532

[170] J. Lee , Y. Jung , M. Lee , J. S. Hwang , J. Guo , W. Shin , J. Min , K. R. Pyun , H. Lee , Y. Lee , Nanoscale Horiz. 2022, 7, 1054. 35775456 10.1039/d2nh00166g

[171] Z. Zhang , L. Wen , L. Jiang , Nat. Rev. Mater. 2021, 6, 622.

[172] Y. Zhou , L. Jiang , Joule 2020, 4, 2244.

[173] T. B. Schroeder , A. Guha , A. Lamoureux , G. VanRenterghem , D. Sept , M. Shtein , J. Yang , M. Mayer , Nature 2017, 552, 214. 29239354 10.1038/nature24670 PMC6436395

[174] J. Feng , M. Graf , K. Liu , D. Ovchinnikov , D. Dumcenco , M. Heiranian , V. Nandigana , N. R. Aluru , A. Kis , A. Radenovic , Nature 2016, 536, 197. 27409806 10.1038/nature18593

[175] M. Macha , S. Marion , V. V. Nandigana , A. Radenovic , Nat. Rev. Mater. 2019, 4, 588.

[176] Y. Tao , Q. Liu , J. Chen , B. Wang , Y. Wang , K. Liu , M. Li , H. Jiang , Z. Lu , D. Wang , Environ. Sci. Technol. 2016, 50, 7889. 27294591 10.1021/acs.est.6b00648

[177] X. Wu , Y. Qiao , Z. Shi , W. Tang , C. M. Li , ACS Appl. Mater. Interfaces 2018, 10, 11671. 29557635 10.1021/acsami.7b19826

[178] B. R. Sreelekshmy , A. J. Rajappan , R. Basheer , V. Vasudevan , A. Ratheesh , M. S. Meera , C. V. Geethanjali , S. M. A. Shibli , ACS Appl. Bio Mater. 2020, 3, 6224. 10.1021/acsabm.0c00753 35021755

[179] Y. Yang , D. Ye , J. Li , X. Zhu , Q. Liao , B. Zhang , Int. J. Hydrogen Energy 2015, 40, 11983.

[180] A. J. Slate , K. A. Whitehead , D. A. Brownson , C. E. Banks , Renewable Sustainable Energy Rev. 2019, 101, 60.

[181] L. Qu , Q. Gou , J. Deng , Y. Zheng , M. Li , Langmuir 2024, 40, 6601. 38478901 10.1021/acs.langmuir.3c03679

[182] J. Mei , T. Liao , H. Peng , Z. Q. Sun , Small Methods 2022, 6, 2101076. 10.1002/smtd.202101076 34954906

[183] J. Ren , Q. Liu , Y. Pei , Y. Wang , S. Yang , S. Lin , W. Chen , S. Ling , D. L. Kaplan , Adv. Mater. Technol. 2021, 6, 2001301.

[184] X. Guo , J. Zhang , L. Yuan , B. Xi , F. Gao , X. Zheng , R. Pan , L. Guo , X. An , T. Fan , Adv. Energy Mater. 2023, 13, 2204376.

[185] M. Fu , W. Chen , Y. Lei , H. Yu , Y. Lin , M. Terrones , Adv. Mater. 2023, 35, 2300940. 10.1002/adma.202300940 36921960

[186] H. J. Sim , C. Choi , D. Y. Lee , H. Kim , J.‐H. Yun , J. M. Kim , T. M. Kang , R. Ovalle , R. H. Baughman , C. W. Kee , Nano Energy 2018, 47, 385.

[187] Y. Yang , Q. Huang , L. Niu , D. Wang , C. Yan , Y. She , Z. Zheng , Adv. Mater. 2017, 29, 1606679. 10.1002/adma.201606679 28234421

[188] T. Xu , Q. Song , K. Liu , H. Liu , J. Pan , W. Liu , L. Dai , M. Zhang , Y. Wang , C. Si , Nano‐Micro Lett. 2023, 15, 98. 10.1007/s40820-023-01073-x PMC10086089 37038023

[189] W. Zhang , S. Ma , X. Ji , X. Liu , Y. Cong , L. Shi , Nat. Electron. 2024, 7, 954.

[190] A. Doricchi , C. M. Platnich , A. Gimpel , F. Horn , M. Earle , G. Lanzavecchia , A. L. Cortajarena , L. M. Liz‐Marzán , N. Liu , R. Heckel , ACS Nano 2022, 16, 17552. 36256971 10.1021/acsnano.2c06748 PMC9706676

[191] C. D. Schuman , S. R. Kulkarni , M. Parsa , J. P. Mitchell , P. Date , B. Kay , Nat. Comput. Sci. 2022, 2, 10. 38177712 10.1038/s43588-021-00184-y

[192] S. Wang , S. Gao , C. Tang , E. Occhipinti , C. Li , S. Wang , J. Wang , H. Zhao , G. Hu , A. Nathan , Nat. Commun. 2024, 15, 4671. 38821961 10.1038/s41467-024-48908-8 PMC11143376

[193] M. Davies , N. Srinivasa , T.‐H. Lin , G. Chinya , Y. Cao , S. H. Choday , G. Dimou , P. Joshi , N. Imam , S. L. Jain , IEEE Micro 2018, 38, 82.

[194] P. A. Merolla , J. V. Arthur , R. Alvarez‐Icaza , A. S. Cassidy , J. Sawada , F. Akopyan , B. L. Jackson , N. Imam , C. Guo , Y. Nakamura , Science 2014, 345, 668. 25104385 10.1126/science.1254642

[195] C. Li , M. Hu , Y. Li , H. Jiang , N. Ge , E. Montgomery , J. Zhang , W. Song , N. Dávila , C. E. Graves , Nat. Electron. 2018, 1, 52.

[196] Y. Chai , Nature 2020, 579, 32. 32132685 10.1038/d41586-020-00592-6

[197] J. Liu , J. Fan , Z. Zhong , H. Qiu , J. Xiao , Y. Zhou , Z. Zhu , G. Dai , N. Wang , Q. Liu , IEEE Trans. Biomed. Circuits Syst. 2023, 17, 952. 37192039 10.1109/TBCAS.2023.3276782

[198] K. Roy , A. Jaiswal , P. Panda , Nature 2019, 575, 607. 31776490 10.1038/s41586-019-1677-2

[199] N. Goldman , P. Bertone , S. Chen , C. Dessimoz , E. M. LeProust , B. Sipos , E. Birney , Nature 2013, 494, 77. 23354052 10.1038/nature11875 PMC3672958

[200] G. M. Church , Y. Gao , S. Kosuri , Science 2012, 337, 1628. 22903519 10.1126/science.1226355

[201] B. Liu , F. Wang , C. Fan , Q. Li , Adv. Mater. 2025, 37, 2412926. 10.1002/adma.202412926 39910849

[202] Y. Erlich , D. Zielinski , Science 2017, 355, 950. 28254941 10.1126/science.aaj2038

[203] L. Organick , S. D. Ang , Y.‐J. Chen , R. Lopez , S. Yekhanin , K. Makarychev , M. Z. Racz , G. Kamath , P. Gopalan , B. Nguyen , Nat. Biotechnol. 2018, 36, 242. 29457795 10.1038/nbt.4079

[204] M. R. Hasan , F. Tabassum , Z. Afrin , in 2023 26th International Conference on Computer and Information Technology (ICCIT) , IEEE, Cox's Bazar, Bangladesh 2023, pp. 1–4.

[205] M. Cui , C. Zhang , Y. Chen , Z. Zhang , T. Wu , H. Wen , IEEE Access 2021, 9, 18052.

[206] K. Li , X. Lu , J. Liao , H. Chen , W. Lin , Y. Zhao , D. Tang , C. Li , Z. Tian , Z. Zhu , Proc. Natl. Acad. Sci. USA 2024, 121, 2410164121. 10.1073/pnas.2410164121 PMC11348301 39145927

[207] C. Zhang , R. Wu , F. Sun , Y. Lin , Y. Liang , J. Teng , N. Liu , Q. Ouyang , L. Qian , H. Yan , Nature 2024, 634, 824. 39443776 10.1038/s41586-024-08040-5 PMC11499255

[208] J. L. Banal , T. R. Shepherd , J. Berleant , H. Huang , M. Reyes , C. M. Ackerman , P. C. Blainey , M. Bathe , Nat. Mater. 2021, 20, 1272. 34112975 10.1038/s41563-021-01021-3 PMC8564878

[209] M. Liu , S. Wang , L. Jiang , Nat. Rev. Mater. 2017, 2, 17036.

[210] U. G. Wegst , H. Bai , E. Saiz , A. P. Tomsia , R. O. Ritchie , Nat. Mater. 2015, 14, 23. 25344782 10.1038/nmat4089

[211] X. Zhao , X. Chen , H. Yuk , S. Lin , X. Liu , G. Parada , Chem. Rev. 2021, 121, 4309. 33844906 10.1021/acs.chemrev.0c01088 PMC9217625

[212] H. Yuk , J. Wu , X. Zhao , Nat. Rev. Mater. 2022, 7, 935.

[213] Z. Xu , H. Chen , H.‐B. Yang , X. Yao , H. Qin , H.‐P. Cong , S.‐H. Yu , Nat. Commun. 2025, 16, 400. 39755695 10.1038/s41467-024-55677-x PMC11700098

[214] D. Madhav , B. Buffel , P. Moldenaers , F. Desplentere , V. Vandeginste , Prog. Mater. Sci. 2023, 139, 101168.

[215] Y. H. Song , K. J. Wu , T. W. Zhang , L. L. Lu , Y. Guan , F. Zhou , X. X. Wang , Y. C. Yin , Y. H. Tan , F. Li , Adv. Mater. 2019, 31, 1905711. 10.1002/adma.201905711 31693256

[216] W. Li , R. Zhou , Y. Ouyang , Q. Guan , Y. Shen , E. Saiz , M. Li , X. Hou , Small 2024, 20, 2401859. 10.1002/smll.202401859 39031996

[217] M. Li , Q. Xu , X. Wu , W. Li , W. Lan , L. Heng , J. Street , Z. Xia , ACS Appl. Mater. Interfaces 2018, 10, 26787. 30020766 10.1021/acsami.8b08501

[218] M. Li , W. Li , Q. Guan , X. Dai , J. Lv , Z. Xia , W.‐J. Ong , E. Saiz , X. Hou , ACS Nano 2021, 15, 19194. 34797635 10.1021/acsnano.1c03882

[219] G. Lu , R. Ma , Y. Zhao , D. Wang , W. Shang , H. Chen , S. A. Khan , M. Li , E. Saiz , Nat. Commun. 2025, 16, 7754. 40835617 10.1038/s41467-025-63123-9 PMC12368072

[220] Y. Wang , J. Zhu , A. Chen , X. Guo , H. Cui , Z. Chen , Y. Hou , Z. Huang , D. Wang , G. Liang , Adv. Mater. 2023, 35, 2303165. 10.1002/adma.202303165 37493625

[221] J. Chen , H. Zeng , Langmuir 2022, 38, 12999. 36260819 10.1021/acs.langmuir.2c02372

[222] M. Li , W. Li , W. Cai , X. Zhang , Z. Wang , J. Street , W.‐J. Ong , Z. Xia , Q. Xu , Mater. Horiz. 2019, 6, 703.

[223] B. Li , P.‐F. Cao , T. Saito , A. P. Sokolov , Chem. Rev. 2022, 123, 701. 36577085 10.1021/acs.chemrev.2c00575

[224] N. Zhu , Q. Teng , Y. Xing , X. Wang , Z. Zhang , X. Wan , Nano Lett. 2024, 24, 12442. 39316758 10.1021/acs.nanolett.4c02852

[225] T. Matsuda , R. Kawakami , R. Namba , T. Nakajima , J. P. Gong , Science 2019, 363, 504. 30705187 10.1126/science.aau9533

[226] M. Li , C. Li , B. R. Blackman , S. Eduardo , Int. Mater. Rev. 2022, 67, 658.

[227] C. Li , M. Li , Z. Ni , Q. Guan , B. R. Blackman , E. Saiz , J. R. Soc., Interface 2021, 18, 20210162. 34129792 10.1098/rsif.2021.0162 PMC8205534

[228] M. Li , S. Zhou , Q. Guan , W. Li , C. Li , F. Bouville , H. Bai , E. Saiz , ACS Appl. Mater. Interfaces 2022, 14, 46077. 36169925 10.1021/acsami.2c13857 PMC9562273

[229] L. Wang , Y. Zhao , Y. Tian , L. Jiang , Angew. Chem. 2015, 127, 14945.

[230] J. Wei , J. Zhang , X. Cao , J. Huo , X. Huang , J. Zhang , Nat. Commun. 2023, 14, 2862. 37208369 10.1038/s41467-023-38678-0 PMC10198997

[231] M. Liao , W. Xu , Y. Song , Z. Pan , H. Zheng , Y. Li , X. Qin , L. Wang , J. Lu , Z. Wang , Nano Energy 2023, 116, 108831.

[232] J. Wang , L. Sun , M. Zou , W. Gao , C. Liu , L. Shang , Z. Gu , Y. Zhao , Sci. Adv. 2017, 3, 1700004. 10.1126/sciadv.1700004 PMC5457146 28630920

[233] Z. Dai , M. Lei , S. Ding , Q. Zhou , B. Ji , M. Wang , B. Zhou , Exploration 2024, 4, 20230046. 38855620 10.1002/EXP.20230046 PMC11022629

[234] M. Lei , K. Feng , S. Ding , M. Wang , Z. Dai , R. Liu , Y. Gao , Y. Zhou , Q. Xu , B. Zhou , ACS Nano 2022, 16, 12620. 35856940 10.1021/acsnano.2c04188

[235] Y. Roh , D. Lim , M. Kang , J. Cho , S. Han , S. H. Ko , Adv. Eng. Mater. 2024, 26, 2400563.

[236] I. Ha , M. Kim , K. K. Kim , S. Hong , H. Cho , J. Kwon , S. Han , Y. Yoon , P. Won , S. H. R. Ko , Adv. Sci. 2021, 8, 2102536. 10.1002/advs.202102536 PMC8529442 34449132

[237] B. Park , J. H. Shin , J. Ok , S. Park , W. Jung , C. Jeong , S. Choy , Y. J. Jo , T.‐i. Kim , Science 2022, 376, 624. 35511972 10.1126/science.abj9912

[238] Y. Roh , M. Kim , S. M. Won , D. Lim , I. Hong , S. Lee , T. Kim , C. Kim , D. Lee , S. Im , Sci. Rob. 2021, 6, abi6774.

[239] S. Zhou , F. Lin , Q. Guan , L. Ding , E. Saiz , M. Li , Device 2025, 3, 100722.

[240] N. J. Sinha , M. G. Langenstein , D. J. Pochan , C. J. Kloxin , J. G. Saven , Chem. Rev. 2021, 121, 13915. 34709798 10.1021/acs.chemrev.1c00712

[241] M. Chen , M. Gao , L. Bai , H. Zheng , H. J. Qi , K. Zhou , Adv. Mater. 2023, 35, 2209566. 10.1002/adma.202209566 36461147

[242] M. Maroto‐Gómez , M. Malfaz , Á. Castro‐González , M. Á. Salichs , IEEE Access 2024, 12, 180146.

[243] D. Boufidis , R. Garg , E. Angelopoulos , D. K. Cullen , F. Vitale , Nat. Commun. 2025, 16, 1861. 39984447 10.1038/s41467-025-57016-0 PMC11845577

[244] A. P. K. Vasconcelos , S. C. Millik , A. Vazquez , N. Sadaba , S. Sateesh , S. Daily , S. Yu , M. Zhang , N. Manitsirisuk , A. Nelson , Annu. Rev. Mater. Res. 2025, 55, 491.

[245] S. C.‐y Shen , M. J. Buehler , Commun. Eng. 2022, 1, 37.

[246] G. Zhou , J. Li , Q. Song , L. Wang , Z. Ren , B. Sun , X. Hu , W. Wang , G. Xu , X. Chen , Nat. Commun. 2023, 14, 8489. 38123562 10.1038/s41467-023-43944-2 PMC10733375

